# Fungal Community in Antarctic Soil Along the Retreating Collins Glacier (Fildes Peninsula, King George Island)

**DOI:** 10.3390/microorganisms8081145

**Published:** 2020-07-29

**Authors:** Juliana Aparecida dos Santos, Edenilson Meyer, Lara Durães Sette

**Affiliations:** 1 Department of General and Applied Biology, Biosciences Institute, São Paulo State University (UNESP), 24A, 1515, Rio Claro 13506-900, SP, Brazil; julibio7@hotmail.com; 2 Department of Microbiology, Immunology and Parasitology, Biological Sciences Center, Federal University of Santa Catarina, Florianopolis 88040-900, SC, Brazil; meyer@ufsc.br

**Keywords:** extremophiles, fungal diversity, glacial soil, Antarctic microbiology, glacier retraction

## Abstract

Glacial retreat is one of the most conspicuous signs of warming in Antarctic regions. Glacier soils harbor an active microbial community of decomposers, and under the continuous retraction of glaciers, the soil starts to present a gradient of physical, chemical, and biological factors reflecting regional changes over time. Little is known about the biological nature of fungi in Antarctic glacier soils. In this sense, this work aimed at studying the behavior of fungal community structure from samples of glacier soil collected after glacial retreat (Collins Glacier). A total of 309 fungi distributed in 19 genera were obtained from eleven soil samples. Representatives of the genera *Pseudogymnoascus* (Ascomycota) and *Mortierella* (Mortierellomycota) were the most abundant isolates in all samples. The data revealed the presence of filamentous fungi belonging to the phylum Basidiomycota, rarely found in Antarctica. Analysis of the generalized linear models revealed that the distance from the glacier as well as phosphorus and clay were able to modify the distribution of fungal species. Environmental variations proved to have influenced the genera *Pseudogymnoascus* and *Pseudeutorium*.

## 1. Introduction

Microbial diversity of the terrestrial Antarctic environment exists mainly in ice-free areas, which have been altered by climate change, representing less than 1% of the continent. The Antarctic Peninsula is the most affected region, where the warming rate is twice the rate of other Antarctic regions [1,2,3,4]. Considering its rate of glacier retreat, Collins Glacier is predicted to disappear in 285 years [5,6,7].

Collins Glacier, commonly referred to as the Bellinghausen Dome, is a small ice dome centered approximately at 62°12′ S latitude, 58°57′ W longitude (area of 15 square km and a maximum altitude of 270 m). Additionally, studies carried out in 2007 involving the radiocarbon dating of moss adjacent to the glacier indicated that the ice cap has been located at or behind its position for much of the last 3500 years [8]. Although the recent literature provides information related to the slow response of the Collins Glacier to climate change, data collected by Simões et al. [7] indicated that the retreat is a consequence of regional warming. Increase in ice-free area drastically modifies biodiversity due to changes in the environment and the sharing of species with other areas [9,10,11].

Microorganisms are transported by terrestrial dust and in precipitation become embedded in the ice formed from falling snow, which can be considered as an excellent matrix for the long-term preservation of these groups of organisms, allowing for the study of both present and ancient microbial diversity [12]. The ice matrix may contain spores and mycelial fragments of fungi, present in the air from thousands of years ago [12,13]. Terrestrial ecosystems covered with ice are being exposed with the retreating of glaciers, which allows a new environment for microorganisms to establish, and provides a great habitat to study the succession of microbial community and its associations with soil nutrients exposed over the years [14,15,16].

Time since deglaciation affects the fungal community composition. Considering the disturbance caused by climate change, species interactions are impaired and force species adaptations, migration, and extinction [14,17,18,19,20], indicating that air temperature in Antarctica is a significant factor for microbial community composition. According to Newsham et al. [20], global warming can lead to about a 20–27% increase in fungal species richness in the southernmost soils by 2100. This change in the composition of fungus community can trigger substantial changes in nutrient cycling and productivity of Antarctic soils. Considering the premise that global warming is directly related to glacial retreat, studies of microbial succession have become relevant.

Rapid environmental warming is a threat to the integrity of ice-influenced ecosystems, where microorganisms are the dominant form of life [21]. Despite the importance of microorganisms in Antarctic soils and the fast warming of the Antarctic Peninsula, little is known about how these microbial communities respond to environmental changes generated by the global warming process [22]. In this context, this study is based on the following objectives: (i) to isolate and identify filamentous fungi from soil samples of glacier retreat (Collins Glacier, Fildes Peninsula, King George Island); (ii) determine whether fungal succession occurs in soils and describe diversity distribution, and (iii) determine whether the composition of the fungal community is associated with the composition of the soil.

## 2. Methods

### 2.1. Sampling Site

**Figure 1:**
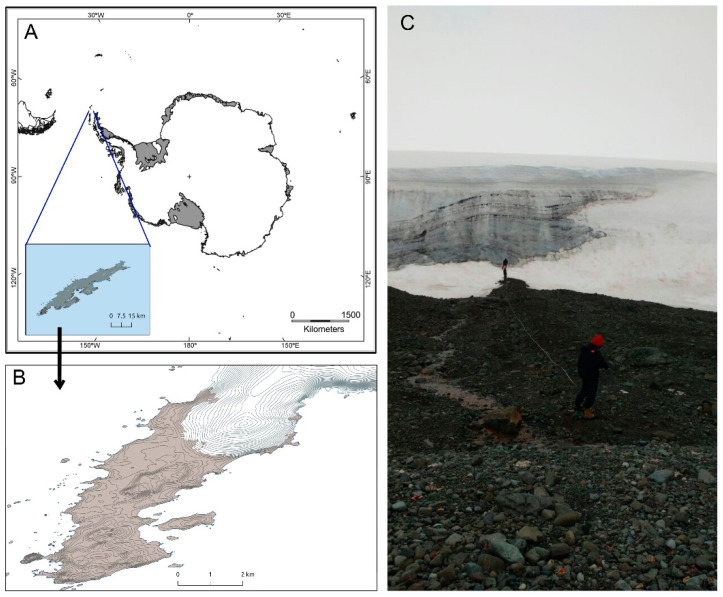
Collins Glacier (Fildes Peninsula, South Shetlands Archipelago, King George Island, Maritime Antarctica). (**A**,**B**) Map of the sampling region; (**C**) sampling.

Soil samples were collected during the XXXIII Brazilian Antarctic Expedition (February 2015) at different points in the foreland of the retreating Collins Glacier (62°10′ S, 58°55′ W), located in the Fildes Peninsula, King George Island, Maritime Antarctica (Figure 1). A total of eleven samples were collected in the transects at points 0, 3, 50, 100, 150, 200, 250, 300, 350, 400, and 800 m from the glacier front (Table S1). The samples were stored in sterile plastic bags and maintained under refrigeration. For each sampling site, three subsamples were collected and pooled together to produce composite samples, yielding the final samples listed in Table S1.

### 2.2. Physicochemical Analysis

The following physicochemical parameters of the soils were assessed: micronutrient (Cu, Fe, Mn, and Zn), organic matter, P, K, Ca, and Mg concentrations, and pH. The evaluation of clay and silt was carried out by granulometric analysis using the densimeter method (or Buyoucos) based on the sedimentation of the particles that make up the soil [23].

These analyses were carried out at the “Luiz de Queiroz” College of Agriculture (Department of Soil Sciences, University of São Paulo, Brazil), according to the methodology described in Van Raij et al. [24]. Physicochemical parameters of the eleven soil samples are shown in Table S2.

### 2.3. Fungal Isolation

Soil samples (12.5 g) were added to Erlenmeyer flasks containing 112.5 mL of NaCl solution (0.9%), and 200 μL of each sample was serially diluted in a 0.085% NaCl solution. For each sample, 10^−1^ and 10^−3^ dilutions were plated in Petri dishes. The following culture media were used for fungal isolation using the spread-plate method (in g·L^−1^): MA2%: 20 malt extract, 15 agar; PDA: 42 potato dextrose agar; PDA10X: 10X-diluted PDA; and BSA: 15 malt extract, 2 yeast extract, 15 agar, 2 lactic acid (added after autoclaving). Streptomycin (0.01 g·L^−1^) and chloramphenicol (0.1 g·L^−1^) (diluted in alcohol) were added to the culture media in order to avoid bacterial growth. The plates were incubated for two months at 5 °C and 15 °C, and colonies of fungi were purified in the same media used for isolation. Two different methods were applied for fungal preservation: cryopreservation at −80 °C (10% glycerol) and the Castellani method at 4 °C (sterile water). The isolates are currently stored in the research collection of the Laboratory of Environmental and Industrial Mycology (LAMAI) associated with Microbial Resource Center (CRM-UNESP) of the São Paulo State University (Rio Claro, SP, Brazil).

### 2.4. Fungal Identification

Extraction of DNAs from filamentous fungi followed the method described by Lacerda et al. [25]. The internal transcribed spacer (ITS) considered the “barcoding region” for fungi was amplified using the universal primers ITS4 (5′TCCTCCGCTTATTGATATGC3′) and ITS5 (5′GGAAGTAAAAGTCGTAACAAGG3′) [26]. The amplicons were purified using the Exonuclease I and alkaline phosphatase enzymes (Thermo Scientific, Waltham, MA, USA) according to the manufacturer’s protocol. The samples were quantified in a NanoDrop^®^ (Thermo Scientific, MA, USA) and sequenced using the BigDye Terminator^®^ v.3.1 kit (Applied Biosystems, California, USA) in an ABI 3500 sequencer (Applied Biosystems, Foster City, CA, USA). The following sequencing conditions were applied: 95 °C/min followed by 28 cycles at 95 °C/15 s, 50 °C/45 s, and 60 °C/4 min. The generated sequences were assembled into contigs in BioEdit v.7.2.5 [27] and compared to homologous sequences deposited in the NCBI-GenBank database using BLAST. Alignments were obtained independently for each file using MAFFT v.7 [28] and refined with Gblocks v.091b [29] (or manually if necessary). Nucleotide substitution models were selected under separated runs for each dataset using the Akaike Information Criterion (AIC) with a confidence interval of 95% in jModelTest 2 v.2.1.10 [30]. The phylogeny was reconstructed through methods of Bayesian Inference (BI) using MrBayes v.3.2.2 [31]. For each dataset, three heated chains and one cold chain were performed; each run consisted of Markov Chain Monte Carlo (MCMC) sampling for two million generations [32]. Convergence occurred when the standard deviation (SD) of the split frequencies fell below 0.01; the first 25% of MCMC generations were discarded as “burn-in”. The final tree was edited on FigTree v.1.4.0 (http://tree.bio.ed.ac.uk/software/figtree/).

### 2.5. Accession Numbers

The sequences generated were deposited in GenBank under accession numbers MN265889-MN266197.

### 2.6. Structure and Composition of Fungal Communities

Fungal diversity (abundance and species richness) was assessed by using the inverse Simpson (1/D), Shannon, and Chao1 indices. Pearson’s correlation between diversity indices and environmental data was performed and the results were classified according to Schober et al. [33] as negligible (0 to 0.09), weak (0.10 to 0.39), moderate (0.40 to 0.69), strong (0.7 to 0.89), and very strong (>0.9). A dbRDA analysis was carried out to visualize the relationship between environmental data (standardized) and species distribution (Sorensen’s dissimilarity matrix). Variables with VIF (Variance Inflation Factor) higher than 10 were excluded from the dbRDA. Generalized linear models using negative binomial distribution were performed to test the effect of environmental variables on species distribution. Statistical analyses were performed using software R (version 3.6.3) and vegan v.2.5.6, mvabund v.4.1.3, and BiodiversityR v.2.11.3 packages. The SIMPER (similarity percentage) [34] was applied to explore the differences in the composition of the fungal communities and to assess which taxa were primarily responsible for the differences found between groups of samples using Past v. 2.17c.

## 3. Results

### 3.1. Fungi from Collins Glacier Soil Samples

A total of 309 filamentous fungal isolates were recovered from eleven soil samples collected at the Collins Glacier. The isolates were recovered in PDA (30%), MA2% (27%), BSA (23%), and diluted PDA (19%), indicating the applicability of these media for the recovery of cultured Antarctic fungi. The greatest number of fungi was isolated at 15 °C (63%). Some isolates were recovered only at 15 °C (six taxa), and one from the genus *Pholiota* (Basidiomycota) only at 5 °C (Table S4).

Individuals belonging to the genera *Pseudogymnoacus*, *Pseudeurotium*, *Mortierella*, and the order Helotiales were able to grow in all culture media and at both incubation temperatures. A greater number of representatives of the genus *Pseudogymnoascus* was isolated in PDA10X medium (22%) and at 5 °C (38%), of the genus *Pseudeurotium* in MA (50%) at both temperatures (50% in each), of the genus *Mortierella* in PDA (45%) and 15 °C (81%), and Helotiales in PDA and BSA (29% in each) with 57% of the isolated at 5 °C (Table S4).

The following taxa were recovered only in BSA medium: *Passalora* (5 °C), *Pholiota* (5 °C), and *Russulales* (15 °C). *Acremonium* (5 °C and 15 °C), *Gibellulopsis* (15 °C), and *Schizophyllum* (15 °C), isolated only in PDA medium and *Thelebolus* and *Xylaria* only in MA medium and at 15 °C (Table S4).

**Figure 2:**
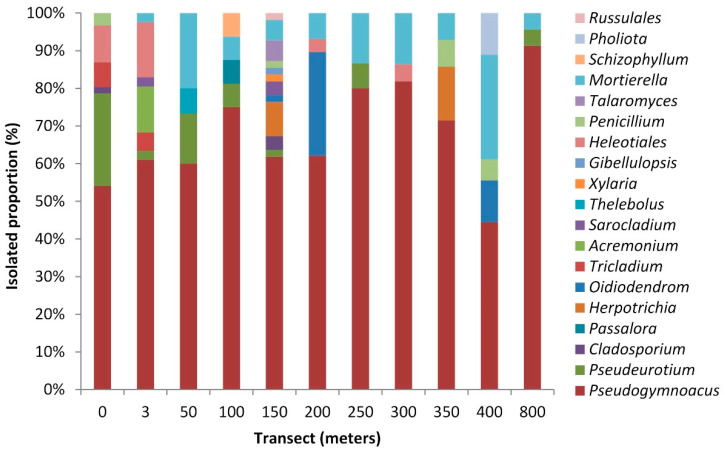
Proportions of filamentous fungal isolates (taxa) in the Collins Glacier retreated soil samples.

Data from sequencing and phylogenetic analyses revealed the predominance of Ascomycota (93.3%), comprising 15 genera, followed by Mortierellomycota (7.3%) with one genus, and Basidiomycota (1.4%) with three genera (Figure 2 and Table S4).

Representatives of Ascomycota were affiliated with the genera *Pseudogymnoascus* (65.0%), *Pseudeutorium* (7.0%), *Oidiodendron* (3.6%), *Herpotrichia* (2.3%), *Tricladium* (1.9%), *Acremonium* (1.6%), *Penicillium* (1.6%), *Cladosporium* (1.0%), *Sarocladium* (1.0%), *Passalora* (0.3%), *Thelebolus* (0.3%), *Xylaria* (0.3%), *Gibellulopsis* (0.3%), and order Helotiales (4.5%).

The Basidiomycota phylum was represented by one isolate identified as belonging to the order *Russulales* (0.3%) and three other isolates from the genera *Schizophyllum* (0.3%) and *Pholiota* (0.6%).

Representatives of the genus *Mortierella* (phylum of Mortierellomycota) showed identity similarity with the species *M. hyalina* (100%), *M. antarctica* (99%), *M. gamsii* (99%)*,* and *M. elongatula* (95%) (Figure 2 and Table S5).

*Pseudogymnoascus* was the dominant genus (65%) with five different species. Some isolates showed similarity with *P. pannorum* (99% identity) and *P. verrucosus* (99% identity). Representatives of *Pseudogymnoascus* were recovered from all samples and greater abundance was observed at the 150 m (17%) and 0 m (16%) points. The genus *Pseudeurotium* was the most abundant at 0 m (68%) represented by two different species, which showed sequence similarity with *P. ovale* (98%) and *P. hygrophilum* (99%). *Oidiodendron* sp. was the most abundant at 200 m (73%) and representatives of the *Tricladium* sp. were found only at 0 and 3 m (Figure 2 and Table S5).

The Dothideomycetes class was represented by *Cladosporium* (100% identity with the species *C. xantochromaticum* and *C. halotolerans,* recovered at 150 and 0 m, respectively), *Passalora* sp. (recovered at 100 m), and *Herpotrichia* sp. (recovered at 150 and 350 m). The *Sordariomycetes* class was represented by *Xylaria* sp., *Sarocladium* sp., *Acremonium* sp., and *Gibellulopis* sp., which were recovered from newly exposed soil samples (0 m) up to 150 m from the glacier retreat, and *Thelebolus* sp., found only at 3 m from the glacier. The *Eurotiomycetes* class was represented by *Penicillium* and *Talaromyces*. *Penicillium* sp., distributed between 0 and 400 m and *Talaromyces* sp. only at 150 m (Figure 2 and Table S4). Most isolates to Helotiales (86%) were recovered at 0 and 3 m, and found up to 300 m from the retreating glacier (Figure 2 and Table S5).

Concerning Basidiomycota, the results revealed the presence of two genera of the order Agaricales (*Schizophyllum* and *Pholiota*) and one representative of the order Russulales (recovered from soil collected at 150 m). *Schizophyllum* sp. showed 99% identity with the specie *S. commune*, obtained at 100 m. The two representatives of *Pholiota* showed 99% identity with *P. baeosperma*, obtained at 400 m.

Considering the number of taxa (Figure 2), the points nearest the glacier (0 and 3 m) showed six and seven genera, respectively. A higher number of isolates (n = 61) was found at 0 m and a higher number of taxa (n = 12) was recovered at 150 m. At 800 m, only the most abundant genera (*Pseudogymnoascus*, *Mortierella,* and *Pseudeurotium*) were found.

Data related to the fungal molecular characterization (sequence similarity and phylogenetic trees) are available in the Supplementary Materials (Table S3 and Figures S1–S15).

### 3.2. Structure and Composition of the Fungal Communities

In general, the 50, 200, 250, 300, 350, and 800 m sites showed less richness expressed in species numbers (S), ranging from 3 to 4. A similar trend was observed for the N (individual numbers) values of the samples at 50, 100, 250, 350, and 400 m (ranging from 14 to 18). In contrast, the samples collected at 200, 300, and 800 m showed N ranging from 22 to 29, while the remaining samples (0, 3, and 150 m) showed N above 40. The highest N and S were found at 0 m and 150 m from the glacier, respectively.

Shannon index (H’), representing richness and dominance, ranged from 0.36 to 1.51. At 150 m, the highest species richness (1.51) and the greatest Chao1 diversity (16.91) were observed (Table 1). This site also presented the largest number of species (S = 12) and the second highest abundance (N = 55). Regarding the major dominance expressed for the Simpson (D) index, represented by 1/λ, the sample collected at 400 m showed the highest value (D = 3.31).

**Figure 3:**
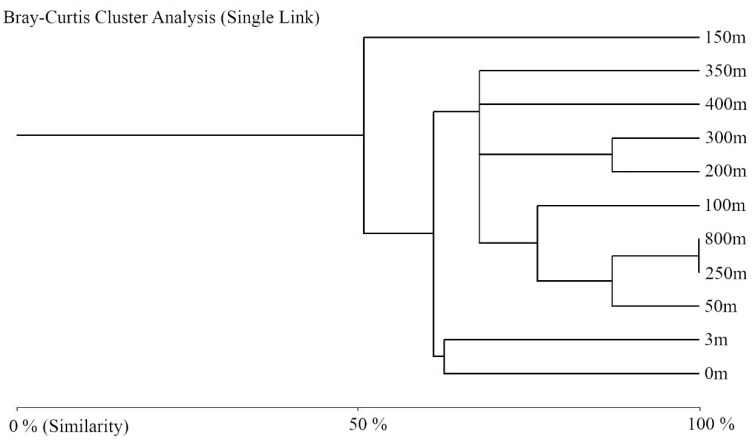
Dendrogram of the Bray–Curtis similarity measures for the filamentous fungi recovered from the Collins Glacier retreat-exposed soil samples.

The similarity between the sampled points was demonstrated in a dendrogram (Figure 3) based on Bray–Curtis distances. It was observed that the sampling sites closest to the glacier (0 and 3 m) formed a cluster and presented greater similarity than the remaining points. The grouping of 250 and 800 m sites showed the same number of taxa (n = 3). The sampling point at 150 m showed a close relationship with the others and is characterized by the greatest richness, the second highest abundance, and highest number of newly emergent taxa. The samples collected at the 200, 300, 400, and 350 m points showed high similarity, with only one taxon differing at 400 m (*Pholiota* sp.).

The results from the SIMPER analysis indicated *Pseudogymnoascus* (18.4% avg. dissimilarity) and *Pseudeutorium* (4.5% avg. dissimilarity) as the taxa that most contributed to generate the differences. These taxa combined accounted for 42% and 10% of the differences, respectively, while the remaining 17 genera were responsible for 8 to 0.5% of the differences.

The results from the generalized linear models (GLM) revealed that the distance from the glacier as well as P and clay were able to modify the structure of the fungal community (*p* < 0.05) (Table 2).

**Figure 4:**
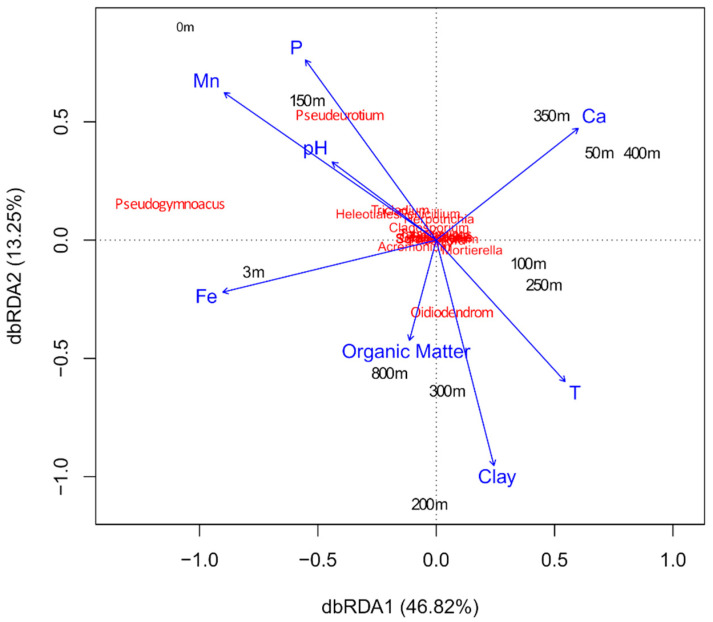
Distance-based redundancy analysis (dbRDA) ordination based on the weighted Sorensen distance with plotting of the environmental parameters and the fungal community at each collection point.

The dbRDA analysis showed that the distribution of *Pseudeurotium* spp. was more associated with the highest levels of phosphorus (P) and manganese (Mn) and lower levels of clay, while the distribution of the *Pseudogymnoacus* spp., was more correlated to iron (Fe). The remaining isolates did not correlate to any environmental conditions (Figure 4).

**Figure 5:**
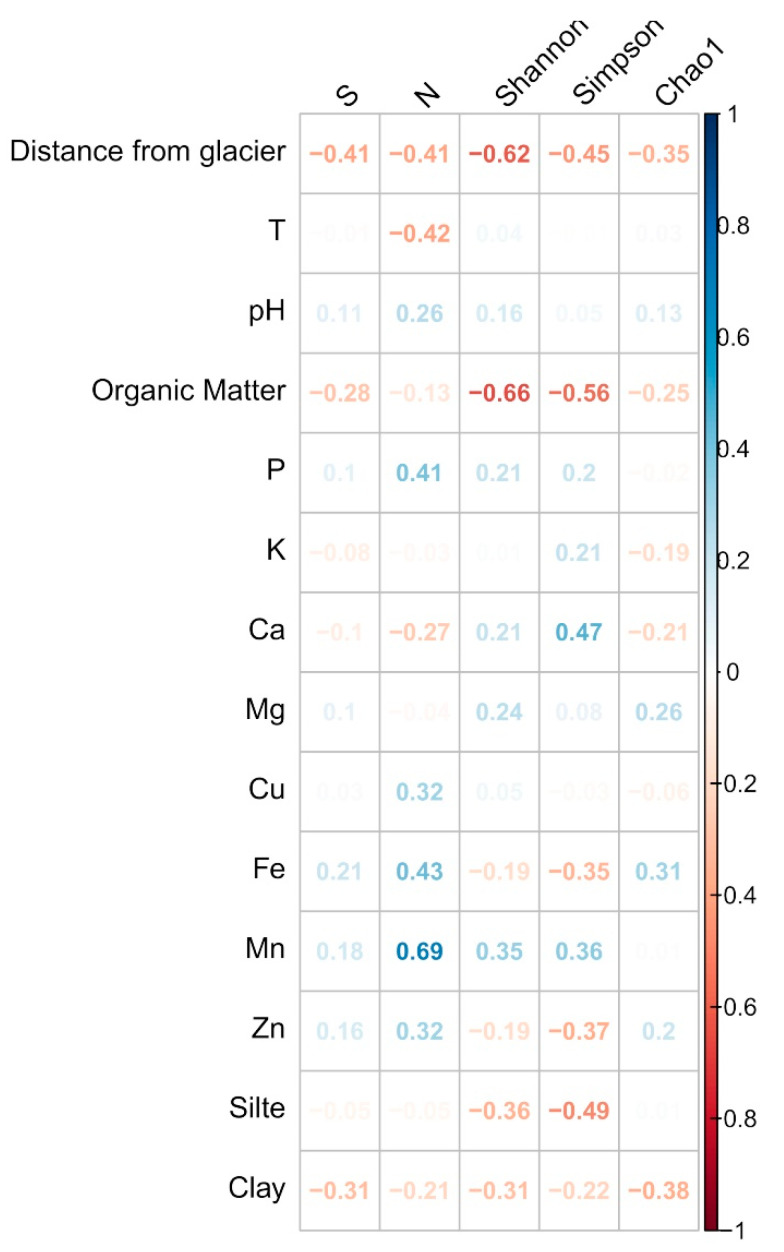
Pearson’s correlation between the diversity indices and environmental data.

The correlation analysis showed that the S correlated negatively (moderate) to the distance from the glacier, while N correlated negatively to the distance from the glacier and temperature, and positively to P, Fe, and Mn. The Shannon index showed a moderate negative correlation with the distance from the glacier and the content of organic matter. The Simpson (D) index correlated negatively to the distance from the glacier, organic matter, and silt, and positively to calcium (Ca) (Figure 5).

## 4. Discussion

Considering that information on microbial structure over time associated with environmental changes is essential to understand soil formation and the evolution of microbial communities, we applied a chronosequence approach in melting the glacier retreats [35,36].

In this study, we recovered a total of 309 filamentous fungi from the 11 Collins Glacier retreated soil samples (collected at 0 to 800 m from the glacier). Filamentous fungi were able to develop at both temperatures applied (5 and 15 °C). The higher number of isolates retrieved from the soil samples at 15 °C possibly resulted from temperature fluctuations in ice-free microhabitats and the higher temperatures observed in maritime Antarctica in relation to the continent [37]. Even though most fungi were retrieved in PDA, MA2%, and BSA media, 19% of the fungi were isolated in PDA10X diluted medium. Interestingly, the representative of the genus *Thelebolus* was recovered only in the diluted medium.

Most of the filamentous fungi identified in this study belong to the phylum Ascomycota. Studies based on culture-dependent and independent approaches have reported the prevalence of Ascomycota in marine and terrestrial Antarctic environments [38,39,40,41,42]. The exclusive use of culture-based fungal detection methods can limit knowledge about fungal diversity. These techniques influence fungal abundance measurements since some species readily grow on culture media to present high rates of sporulation. In this sense, some very slow growing psychrophilic fungi could be missed [38]. Additionally, many species of “unculturable” fungi are only detected using molecular techniques [42]. However, previous research on soils from Antarctica using culture-based methods and molecular techniques revealed that approximately 2/3 of total taxa could be detected using culturing-based investigations [38].

Our results highlight the dominance of the genera *Pseudogymnoascus* (Ascomycota), *Mortierella* (Mortierellomycota), and *Pseudeutorium* (Ascomycota). Since having been found in almost the entire chronosequence of glacier retreat from soil freshly exposed to the oldest site, *Pseudogymnoascus* and *Mortierella* probably present high adaptation to the Antarctic environment, which explains their predominance. The genus *Pseudogymnoascus* includes species distributed worldwide [43,44]. Representatives of this taxon are psychrophilic and able to grow at very low temperatures (down to −20 °C) with occurrence reported for the soils of Arctic, alpine, and Antarctic regions [41,45,46]. Moreover, Arenz et al. [38] suggest that this genus is a prevalent taxon in Antarctica and generally has the ability to colonize and utilize different carbon sources, playing a role in decomposition and nutrient cycling in this environment. According to Lorch et al. [43], the diversity of *Pseudogymnoascus* must be higher than previously inferred based on traditional taxonomic methods. Some of the 200 isolates of *Pseudogymnoascus* obtained in this study could represent new species, therefore, further studies should be conducted to elucidate their taxonomic positions. Representatives of the cold-tolerant genus *Mortierella* were isolated at all sites from 3 to 800 m. These results corroborate the previous reports related to the prevalence of *Mortierella* in Antarctica [47,48,49,50]. Fungi belonging to this genus have been isolated from different substrates of Antarctica such as permafrost [51], macroalgal thalli [48], floating wood in seawater [52], mosses [53], rhizosphere of *Deschampsia antarctica*, and *Colobanthus quitensis* [46,54], soil [55], and marine invertebrates [56]. Species representing the genus *Pseudeurotium* are endophytic and have a cosmopolitan distribution [57], in addition to having been reported to be associated with bat hibernacula [43,44,58,59]. Previous studies on fungi diversity in the Antarctic Peninsula reported the presence of representatives of *Pseudeurotium* in soil, wood, and sponges, in addition to marine and lake sediments [41,60,61]. The following less abundant Ascomycota fungi (<5.0%) found in this study have already been reported in Antarctic environments: *Tricladium*, *Oidiodendron*, *Herpotrichia*, *Acremonium*, *Penicillium*, *Talaromyces, Cladosporium*, *Sarocladium,* and *Thelebolus* [41,46,62,63,64,65,66,67,68]. However, it is worth highlighting that according to the literature, representatives of *Passalora, Gibellulopsis,* and *Xylaria* had have never been reported in this environment previously.

Filamentous fungi from the phylum Basidiomycota are decomposers and rarely found in Antarctica. Most of the fungi from this group reported in maritime and continental Antarctica are yeasts [41,46,60]. Fruiting of Basidiomycota (genera *Pholiota* and *Omphalina*) has been reported on Deception Island [69], probably introduced due to human activity. In this study, four isolates belonging to this phylum were found, representing two genera of the orders Agaricales (*Pholiota* and *Schizophyllum*) and Russulales. The literature presents only one report of *Schizophyllum commune* occurrence in the Antarctic environment [70]. The fungal isolates identified in this study belonging to the orders Helotiales and Russulales presented low identity with fungal sequences deposited in the GenBank database, suggesting that these isolates may represent new species or even genera. However, to confirm this possibility, further molecular and conventional taxonomic experiments should be carried out.

Even though anthropogenic influence on the abundance and diversity of fungal species at low temperatures is still scarce [65], some studies have indicated that anthropogenic action in the polar region can change the fungal species composition and lead to the propagation of eurytopic species at low temperature [71] including opportunistic pathogens and degraders [72]. In addition, many fungi associated with points near the glacier were mostly saprophytic, and the most distant points showed dominance of cosmopolitan fungi. These data corroborate with Dresch et al. [73], who found that many fungal taxa might present an alternative saprobial lifestyle in snow-covered areas.

The fungi found throughout the Collins Glacier retreated soils are cosmopolitan. According to Cox et al. [74], endemic and cosmopolitan fungi may have different dispersal strategies and degrees of adaptations to the extreme environment of these soils. Distribution patterns of microbes can influence their abundance in communities. In this study, *Xylaria, Sarocladium, Acremonium*, and *Gibellulopis* (representing the Sordiomycetes class) were recovered from newly exposed soil samples (up to 150 m from the retreating glacier). This result indicates the impermanence of these genera during the primary succession over the years. Species belonging to these genera are known as saprotrophs and pathogens of a number of plants [75].

Data from sequence and phylogenetic analyses revealed fungal communities in different developing soils formed after the retreat of the Collins Glacier. At the points where the soil was freshly exposed and moist due to water flowing from the glacier (near the glacier), the number of taxa varied from four to seven. Snow cover is an excellent thermal insulator and could enable and promote the growth of active soil microbiota. Disregarding the 150 m point, with 12 taxa, the successive sampling points showed a decrease in the number of taxa (n = 3 to 5). At 800 m, where the sample was centuries old, only three taxa were recovered.

Our results indicate that the distance from the glacier is associated with a change in fungal communities (Table 2) with a lower number of species (S) and isolates (N) as well as lower diversity (based on Shannon and Simpson). Such reduced diversity probably results from the dominance of stronger organisms and the selection pressure exerted by higher temperature, altered soil composition, and extreme conditions of the Antarctic environment. According to Trowbridge and Jumpponen [76], successional age is one of the main factors that defines fungal communities and selects the members to establish and survive successfully.

Several atmospheric variables and soil characteristics modified by the weathering process over the years are capable of modifying the community of microorganisms present in these environments. The GLM analysis performed in this study found P and clay to be decisive for the differences in fungal communities. Pearson’s correlation showed a moderate correlation between the indices of wealth and diversity and some environmental variables such as T (°C), organic matter, P, Ca, Fe, Mn, and silt. By applying a structural equation model (SEM), Siciliano et al. [77] verified the effect of the soil nutrients set, in addition to pH, on fungi structure in glacial soil. Canini et al. [42] reported the effects of Ca, Mg, K, pH, silt and clay on the abundance of certain fungi groups in Antarctica soil. Our results did not indicate any influence of pH on fungal community, possibly because this variable does not vary very much between samples (5.6 to 6.1).

Our results revealed important characteristics of the distribution of the cultured fungal community in soils of the retreated Collins Glacier. This habitat shelters a cultivable fungal community with low diversity and richness, mainly represented by Ascomycota and cosmopolitan fungi. Such a low abundance of species may explain the absence of patterns related to environmental characteristics. The genera *Pseudogymnoascus* and *Pseudeutorium* responded to environmental variations corresponding to distance from the glacier, phosphorus, and clay.

The low diversity of the Antarctic ecosystem is a consequence of its extreme conditions, especially low temperatures and water availability [78], which complies with data globally reported for fungi indicating lower richness from the mid-latitudes to the poles [79,80]. In addition to these adversities, other variables including competition between species can shape the microbial community. Our findings highlight the importance of studies on fungal communities in extreme environments, especially in areas that are more susceptible to impacts of global warming.

## Figures and Tables

**Table 1:** Indices and richness estimator (α-diversity) for the correlated sampled points in meters from the retreating Collins Glacier.

Sample (Meters)	S (N) *	Shannon	Inv-Simpson	Chao1
0	6 (61)	1.26	2.71	6
3	7 (41)	1.26	2.43	8.46
50	4 (15)	1.08	2.37	4
100	5 (16)	0.91	1.73	10.63
150	12 (55)	1.51	2.49	16.91
200	4 (29)	0.95	2.14	4
250	3 (15)	0.63	1.51	3
300	3 (22)	0.58	1.45	3
350	4 (14)	0.90	1.85	4.46
400	5 (18)	1.37	3.31	5
800	3 (23)	0.36	1.19	3.96

* Note: S is the total number of fungal species; N is the total number of fungal isolates.

**Table 2:** Analysis of generalized linear models (GLM) between the cultivable fungus community and the environmental variables of the Collins Glacier (Fildes Peninsula, South Shetlands Archipelago, King George Island, Maritime Antarctica).

Variable	*p*-Value
Sample (meters)	0.020
T	0.089
Silt	0.416
Clay	0.049
O.M.	0.277
pH	0.129
P	0.030
K	0.604
Ca	0.188
Mg	0.455
Fe	0.218
Cu	0.257
Mn	0.059

## Supplementary Materials

The following are available online at https://www.mdpi.com/2076-2607/8/8/1145/s1, Table S1. Data for the soil samples collected at Collins Glacier (Fildes Peninsula, South Shetlands Archipelago, King George Island, Maritime Antarctica). Table S2. Physicochemical parameters of the eleven soil samples collected at Collins Glacier (Fildes Peninsula, King George Island, Antarctica). Table S3. Taxonomic affiliation of the Antarctic fungi isolated from samples collected at Collins Glacier (Fildes Peninsula, King George Island, Antarctica). Table S4. Number of filamentous fungi isolated per taxa from the Collins Glacier retreated soil samples using different culture media and two temperature of the incubation. Table S5. Taxonomic affiliations of the filamentous fungi isolated from the Collins Glacier retreated soil samples. Figure S1. Phylogenetic analysis of *Oidiodendron* species isolated in this study, with sequences of the closest species deposited in GenBank. The tree was built based on the ITS region sequences using Bayesian Inference. Figure S2. Phylogenetic analysis of *Herpotrichia* species isolated in this study, with sequences of the closest species deposited in GenBank. The tree was built based on the ITS region sequences using Bayesian Inference. Figure S3. Phylogenetic analysis of *Passalora* species isolated in this study, with sequences of the closest species deposited in GenBank. The tree was built based on the ITS region sequences using Bayesian Inference. Figure S4. Phylogenetic analysis of *Pseudeurotium* species isolated in this study, with sequences of the closest species deposited in GenBank. The tree was built based on the ITS region sequences using Bayesian Inference. Figure S5. Phylogenetic analysis of *Sarocladium* and *Acremonium* species isolated in this study, with sequences of the closest species deposited in GenBank. The tree was built based on the ITS region sequences using Bayesian Inference. Figure S6. Phylogenetic analysis of *Thelebolus* specie isolated in this study, with sequences of the closest species deposited in GenBank. The tree was built based on the ITS region sequences using Bayesian Inference. Figure S7. Phylogenetic analysis of *Xylaria* specie isolated in this study, with sequences of the closest species deposited in GenBank. The tree was built based on the ITS region sequences using Bayesian Inference. Figure S8. Phylogenetic analysis of *Pholiota* species isolated in this study, with sequences of the closest species deposited in GenBank. The tree was built based on the ITS region sequences using Bayesian Inference. Figure S9. Phylogenetic analysis of *Helotiales* order isolated in this study, with sequences of the closest species deposited in GenBank. The tree was built based on the ITS region sequences using Bayesian Inference. Figure S10. Phylogenetic analysis of *Schizophyllum* specie isolated in this study, with sequences of the closest species deposited in GenBank. The tree was built based on the ITS region sequences using Bayesian Inference. Figure S11. Phylogenetic analysis of *Gibellulopsis* specie isolated in this study, with sequences of the closest species deposited in GenBank. The tree was built based on the ITS region sequences using Bayesian Inference. Figure S12. Phylogenetic analysis of Mortierella specie isolated in this study, with sequences of the closest species deposited in GenBank. The tree was built based on the ITS region sequences using Bayesian Inference. Figure S13. Phylogenetic analysis of *Tricladium* specie isolated in this study, with sequences of the closest species deposited in GenBank. The tree was built based on the ITS region sequences using Bayesian Inference. Figure S14. Phylogenetic analysis of *Pseudogymnoascus* specie isolated in this study, with sequences of the closest species deposited in GenBank. The tree was built based on the ITS region sequences using Bayesian Inference. Figure S15. Phylogenetic analysis of *Cladosporium* specie isolated in this study, with sequences of the closest species deposited in GenBank. The tree was built based on the ITS region sequences using Bayesian Inference.
