# Supplementary material for: Fungal Community in Antarctic Soil Along the Retreating Collins Glacier (Fildes Peninsula, King George Island)
Source: Microorganisms. 2020 Jul 29;8(8):1145. doi: 10.3390/microorganisms8081145 (PMC7465374; doi:10.3390/microorganisms8081145)
Supplement: Supplementary file 1 [file microorganisms-08-01145-s001.pdf]

Supplementary Material

Table S1. Data for the soil samples collected at Collins Glacier (Fildes Peninsula, South Shetlands Archipelago, King George Island, Maritime Antarctica).

Site (meters)	Geographic coordinate	Average T (°C)
0	62° 09.821'S 58° 55.373'W	0.9
3	62° 09.821'S 58° 55.377'W	5.1
50	62° 09.821'S 58° 55.373'W	2.7
100	62° 09.804'S 58° 55.506'W	3.9
150	62° 09.794'S 58° 55.560'W	3.6
200	62° 09.790'S 58° 55.599'W	4.9
250	62° 09.782'S 58° 55.658'W	4.5
300	62° 09.776'S 58° 55.712'W	3.5
350	62° 09.766'S 58° 55.771'W	4.5
400	62° 09.753'S 58° 55.825'W	4.5
800	62° 09.674'S 58° 56.249'W	2.5

Table S2. Physicochemical parameters of the eleven soil samples collected at Collins Glacier (Fildes Peninsula, King George Island, Antarctica).

Sample (meters)	T°C	pH	Organic Matter ($g.dm^{-3}$)	P ($mg.dm^{-3}$)	K ($mmol_c.dm^{-3}$)	Ca ($mmol_c.dm^{-3}$)	Mg ($mmol_c.dm^{-3}$)	Cu ($mg.dm^{-3}$)	Fe ($mg.dm^{-3}$)	Mn ($mg.dm^{-3}$)	Zn ($mg.dm^{-3}$)	Silt ($g\ kg^{-1}$)	Clay ($g\ kg^{-1}$)
0 m	0.9	6.1	5	369	11,9	197	101	4.1	67	38.7	0.4	162	52
3 m	5.1	6.1	5	320	9.9	236	110	4.2	64	12.8	0.3	129	52
50 m	2.7	6.1	6	340	7.9	210	125	4.6	45	10.3	0.2	102	82
100 m	3.9	6	4	269	6.7	153	136	2.8	67	4.4	0.4	236	52
150 m	3.6	5.8	7	277	9	170	92	3	66	5.3	0.5	307	78
200 m	4.9	5.7	5	230	7.2	139	103	3.3	54	6.6	0.5	338	156
250 m	4.5	6.1	8	313	8.4	245	98	3.1	35	6.8	0.4	415	78
300 m	3.5	5.7	9	270	6.6	119	81	4.5	77	4	0.6	345	131
350 m	4.5	5.6	5	262	14.8	277	87	2	53	2.3	0.3	181	78
400 m	4.5	5.6	6	257	16.2	470	78	1.6	39	1.7	0.2	125	77
800 m	2.5	5.7	17	274	16.2	266	72	2.2	73	1.5	0.3	174	77

Table S3. Taxonomic affiliation of the Antarctic fungi isolated from samples collected at Collins Glacier (Fildes Peninsula, King George Island, Antarctica).

Fungal identification	BLAST closest relatives	Accession Number	ID (%)	N° ofi solates
Oidiodendron sp.	*Oidiodendrontruncatum*UAMH 1399	NR111036	99	11
*Herpotrichia*sp.	*Herpotrichiajuniperi*TR_Cl_4 18S	HM853976	98	7
Passalora sp.	*Passaloraloranthi*C8a8	JX436780	100	1
Pseudeurotium sp1	*Pseudeurotium ovale* CBS 389.54	NR145346	98	7
*Pseudeurotium*sp2	*Pseudeurotiumhygrophilum*	NR111128	99	15
Acremonium sp.	*Acremoniumcereale* IHBF 2215	MF326602	99	5
Sarocladium sp.	*Sarocladiumimplicatum* CBS 397.70A	MH859761	98	3
*Thelebolus*sp.	*Thelebolusmicrosporus*Ant170	MG586989	100	1
*Xylaria*sp.	*Xylaria multiplex* 1005	KP133436	99	1
Pholiota sp.	*Pholiotabaeosperma* TFB8315	KY559332	99	2
Helotiales sp.	*Helotiales* sp. UFMGCB 12371	MK889360	99	14
Schizophyllum sp.	*Schizophyllumcommune* CBS 579.83	MH861655	99	1
*Gibellulopsis*sp.	*Gibellulopsispiscis*CBS 892.70	NR_145354	99	1
*Mortierella*sp1	*Mortierellagamsii*CBS 749.68	NR_152954	99	3
Mortierella sp2	*Mortierellahyalina*CBS 223.35	NR16354	100	2
Mortierella sp3	*Mortierellaantarctica* CBS 609.70	NR111580	99	8
Mortierella sp4	*Mortierellaelongatula* CBS 488.70	NR111582	95	9
Tricladium sp.	*Tricladiumminutum*	JQ412863	95	6
Cladosporium sp1	*Cladosporiumxantochromaticum* CPC11609	EF679356	100	2
Cladosporium sp2	*Cladosporiumhalotolerans* CBS 130257	MH865505	100	1
Talaromyces sp.	*Talaromycesfuniculosus* CBS 126817	MH864243	100	3
*Penicillium*sp.	*Penicillium*sp. MCCC001	MN481191	100	5
Pseudogymnoascus sp1	*Pseudogymnoascusverrucosus* UAMH 10580	DQ117441	99	2
Pseudogymnoascus sp2	*Pseudogymnoascuspannorum* CBS 127774	MH864770	99	4
Pseudogymnoascus sp3	*Pseudogymnoascus* sp. LW65	MH128228	99	84
Pseudogymnoascus sp4	*Pseudogymnoascuspannorum* UAMH 1030	DQ117436	99	1
Pseudogymnoascus sp5	*Pseudogymnoascus* sp. CCMGE55	MH790447	99	109
**Total**				309

Table S4. Number of filamentous fungi isolated per taxa from the Collins Glacier retreated soil samples, using different culture media and two temperature of the incubation.

	5°C				15°C			
	PDA	PDA10X	MA	BSA	PDA	PDA10X	MA	BSA
Ascomycota								
Pseudogymnoascus	24	14	21	15	30	34	33	29
Pseudeutorium	3	1	3	4	1	1	8	1
Tricladium	2	-	1	-	-	-	3	-
Oidiodendron	1	1	-	1	2	3	-	3
Herpotrichia	2	-	2	-	2	-	1	-
Acremonium	3	-	-	-	1	-	-	-
Penicillium	-	1	1	1	2	-	-	-
Talaromyces	-	-	-	-	1	-	-	2
Cladosporium	-	-	-	1	1	-	-	1
Sarocladium	1	-	-	-	1	-	-	1
Passalora	-	-	-	1	-	-	-	-
Thelebolus	-	-	-	-	-	-	1	-
Xylaria	-	-	-	-	-	-	1	-
Gibellulopsis	-	-	-	-	1	-	-	-
Helotiales	4	1	1	2	1	2	-	3
Basidiomycota								
Schizophyllum	-	-	-	-	1	-	-	-
Pholiota	-	-	-	2	-	-	-	-
Russulales	-	-	-	-	1	-	-	-
Mortierellomycota								
Mortierella	1	-	3	-	10	3	4	1
Total	41	18	32	28	55	44	50	41
	119				190			

Table S5. Taxonomic affiliations of the filamentous fungi isolated from the Collins Glacier retreated soil samples

TAXON	Distancefromglacier (m)											Total	FR (%)[1]
	0	3	50	100	150	200	250	300	350	400	800		
**Ascomycota**													
*Pseudogymnoascus*sp1	-	-	1	1	-	-	-	-	-	-	-	2	0.65
*Pseudogymnoascus*sp2	4	-	-	-	-	-	-	-	-	-	-	4	1.29
*Pseudogymnoascus*sp3	24	23	9	9	6	6	2	-	-	-	5	84	27.18
*Pseudogymnoascus*sp4	-	1	-	-	-	-	-	-	-	-	-	1	0.32
*Pseudogymnoascus*sp5	5	1	-	2	24	12	10	18	10	8	19	109	35.28
*Pseudeutorium*sp1	5	1	-	-	1	-	-	-	-	-	-	7	2.27
*Pseudeutorium*sp2	10	2	-	1	-	-	1	-	-	-	1	15	4.85
Tricladium	4	2	-	-	-	-	-	-	-	-	-	6	1.94
Oidiodendron	-	-	-	-	1	8	-	-	-	2	-	11	3.56
Herpotrichia	-	-	-	-	5	-	-	-	2	-	-	7	2.27
Acremonium	-	5	-	-	-	-	-	-	-	-	-	5	1.62
Penicillium	2	-	-	-	1	-	-	-	1	1	-	5	1.62
Talaromyces	-	-	-	-	3	-	-	-	-	-	-	3	0.97
*Cladosporium*sp1	-	-	-	-	2	-	-	-	-	-	-	2	0.65
*Cladosporium*sp2	1	-	-	-	-	-	-	-	-	-	-	1	0.32
Sarocladium	-	1	-	-	2	-	-	-	-	-	-	3	0.97
Passalora	-	-	-	1	-	-	-	-	-	-	-	1	0.32
Thelebolus	-	-	1	-	-	-	-	-	-	-	-	1	0.32
Xylaria	-	-	-	-	1	-	-	-	-	-	-	1	0.32
Gibellulopsis	-	-	-	-	1	-	-	-	-	-	-	1	0.32
Helotiales	6	6	-	-	-	1	-	1	-	-	-	14	4.53
**Basidiomycota**													
Schizophyllum	-	-	-	1	-	-	-	-	-	-	-	1	0.32
Pholiota	-	-	-	-	-	-	-	-	-	2	-	2	0.65
Russulales	-	-	-	-	1	-	-	-	-	-	-	1	0.32
**Mortierellomycota**													
*Mortierella*sp1	-	-	-	-	1	-	-	-	-	2	-	3	0.97
*Mortierella*sp2	-	-	1	-	-	-	-	-	-	-	1	2	0.65
*Mortierella*sp3	-	-	2	-	-	-	2	3	-	-	1	8	2.59
*Mortierella*sp4	-	-	-	1	-	2	2	-	2	2	-	9	2.91
Total	61	41	15	16	55	29	15	22	14	18	23	**309**	**100**

[1] FR (%) = Relative frequency of isolate.

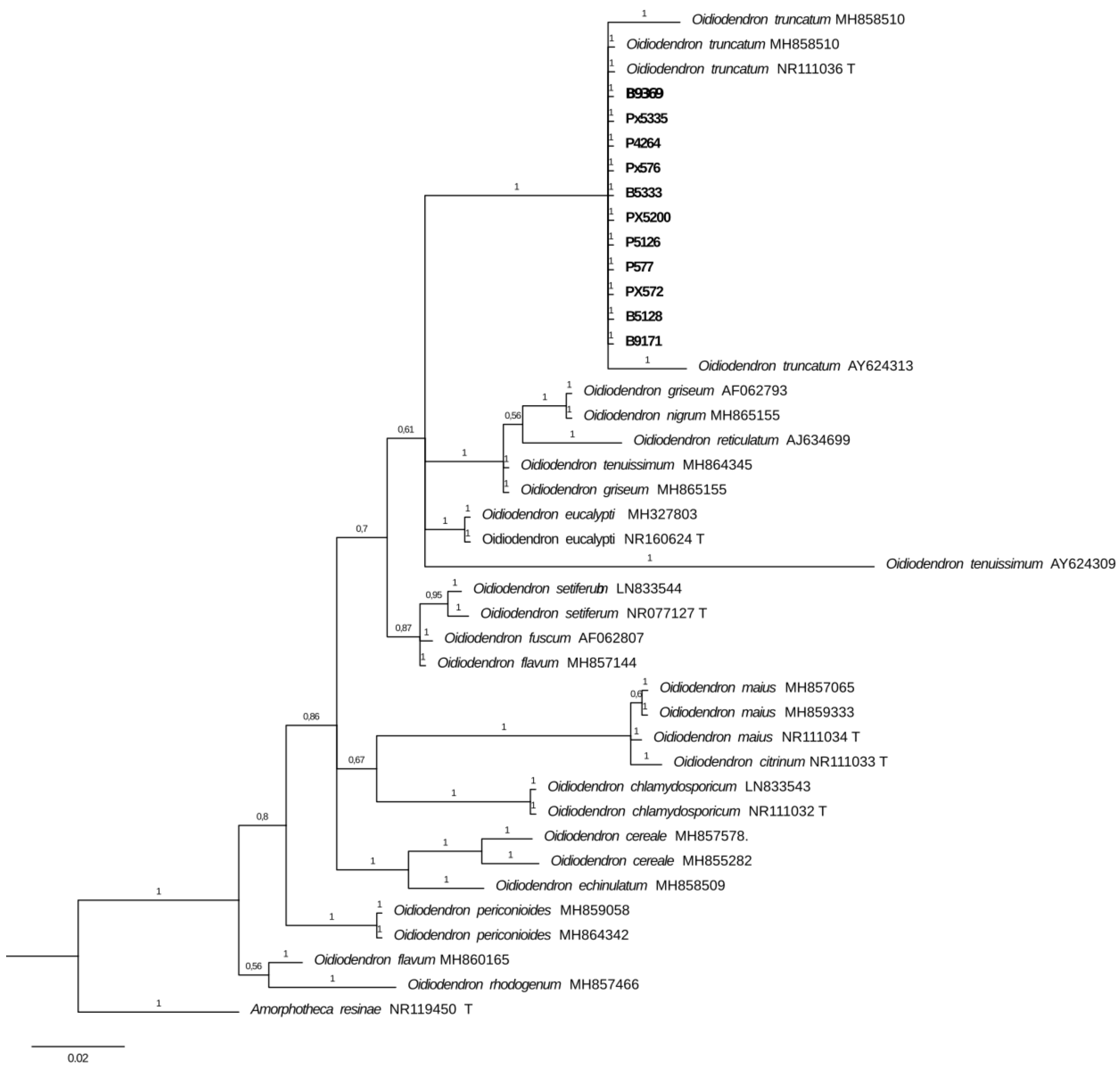

Figure S1. Phylogenetic analysis of *Oidiodendron* species isolated in this study, with sequences of the closest species deposited in GenBank. The tree was built based on the ITS region sequences using Bayesian Inference.

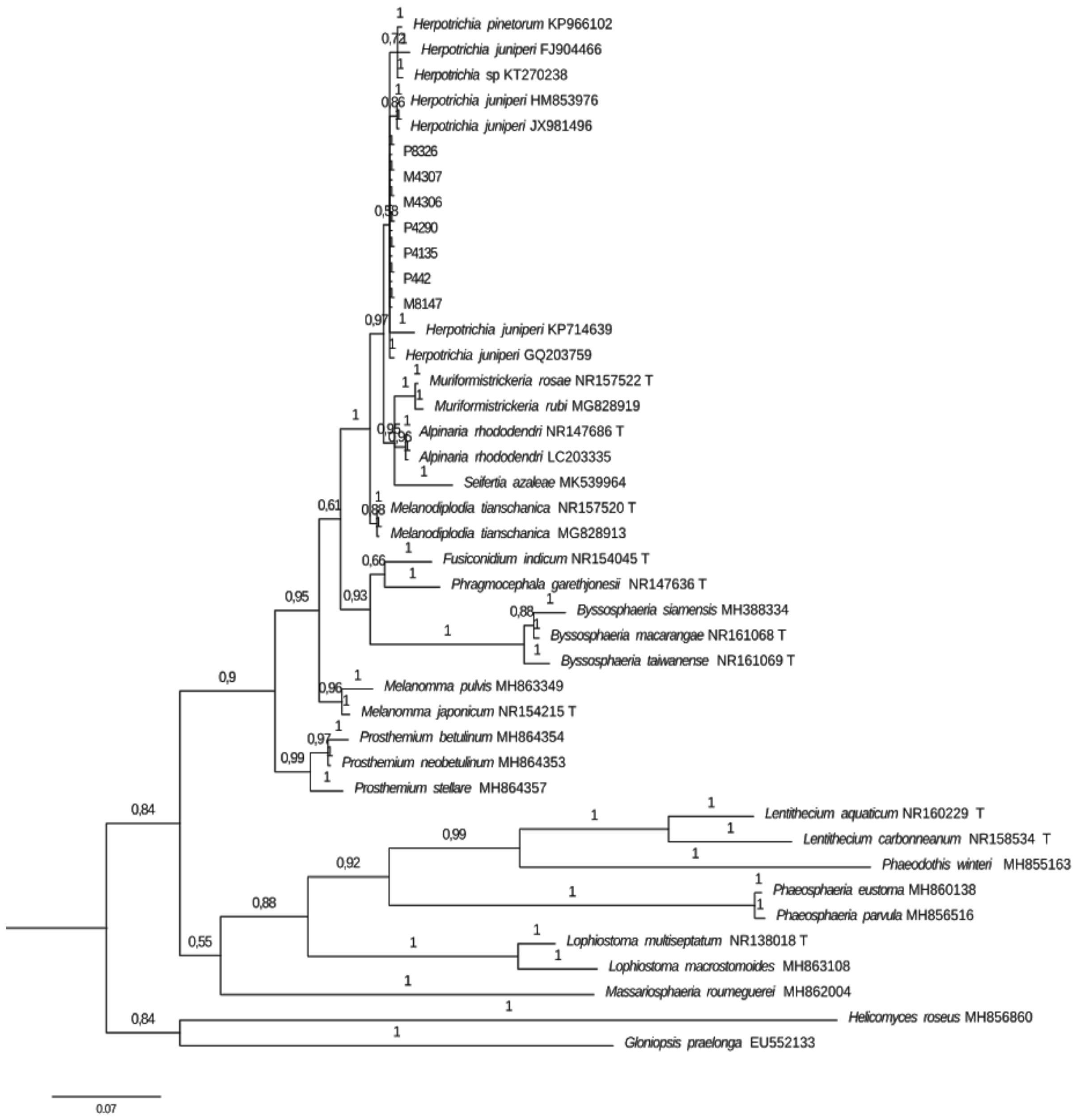

Figure S2. Phylogenetic analysis of *Herpotrichia* species isolated in this study, with sequences of the closest species deposited in GenBank. The tree was built based on the ITS region sequences using Bayesian Inference.

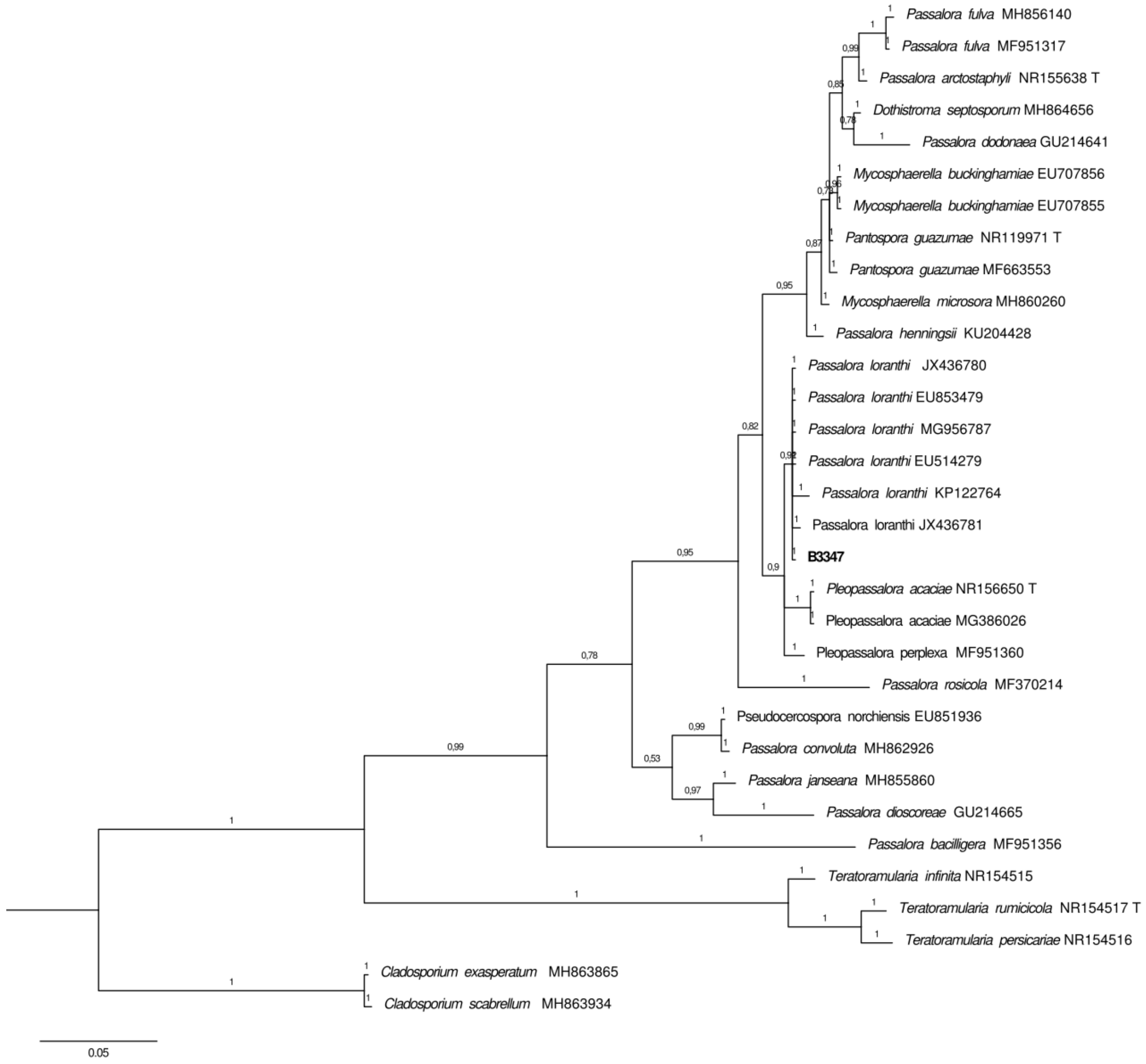

Figure S3. Phylogenetic analysis of *Passalora* species isolated in this study, with sequences of the closest species deposited in GenBank. The tree was built based on the ITS region sequences using Bayesian Inference.

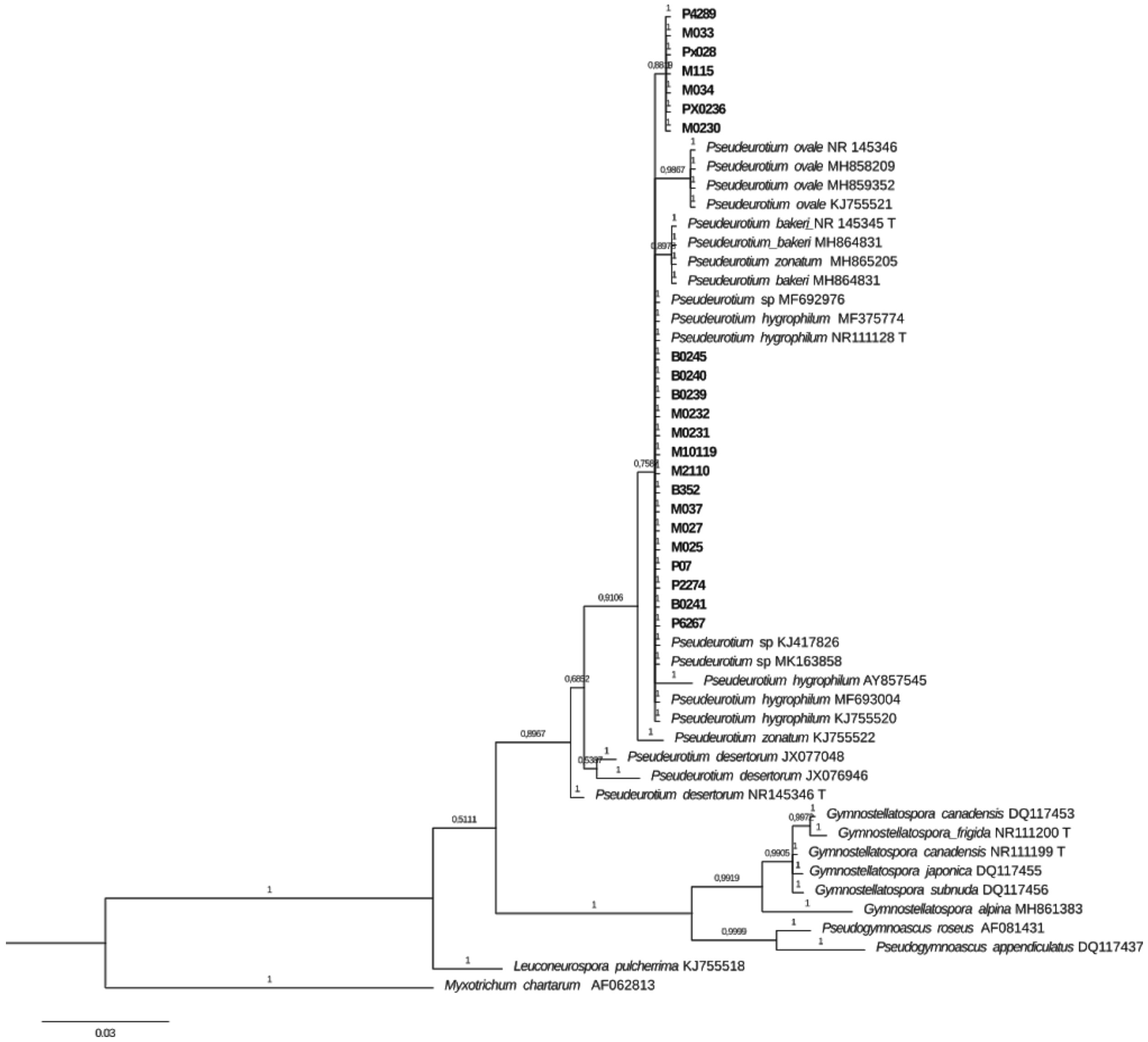

Figure S4. Phylogenetic analysis of *Pseudeurotium* species isolated in this study, with sequences of the closest species deposited in GenBank. The tree was built based on the ITS region sequences using Bayesian Inference.

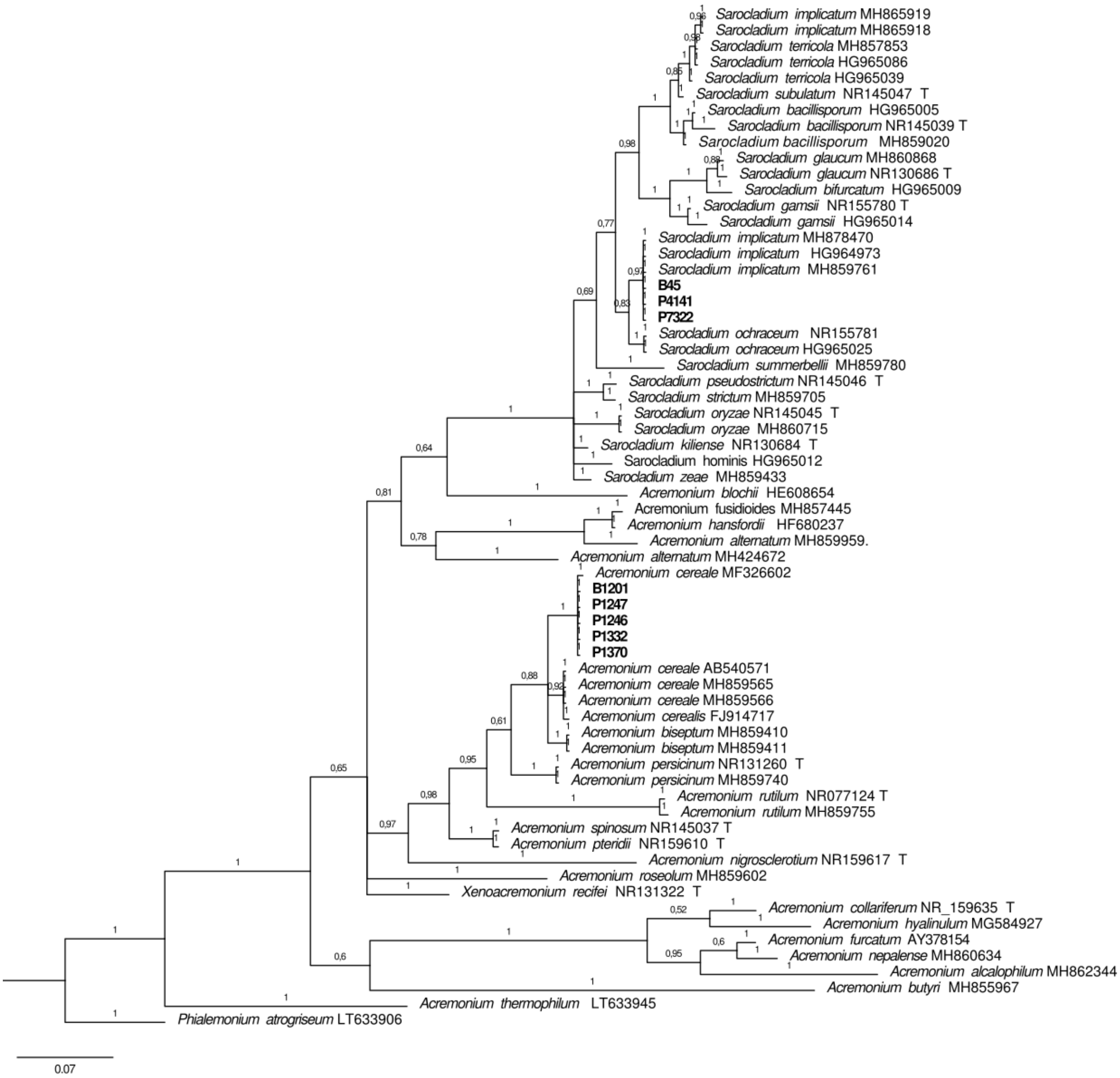

Figure S5. Phylogenetic analysis of *Sarocladium* and *Acremonium* species isolated in this study, with sequences of the closest species deposited in GenBank. The tree was built based on the ITS region sequences using Bayesian Inference.

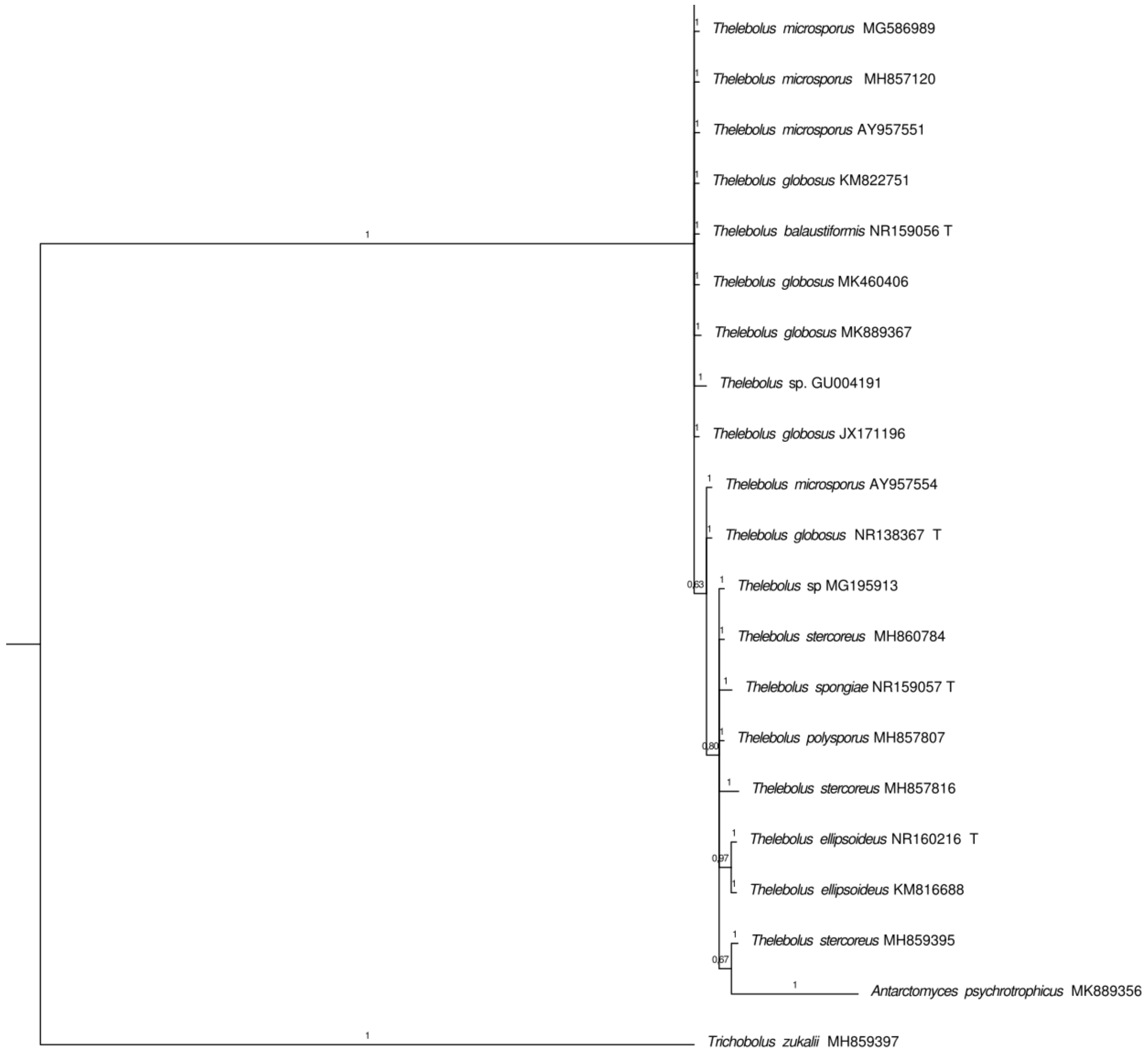

Figure S6. Phylogenetic analysis of *Thelebolus* species isolated in this study, with sequences of the closest species deposited in GenBank. The tree was built based on the ITS region sequences using Bayesian Inference.

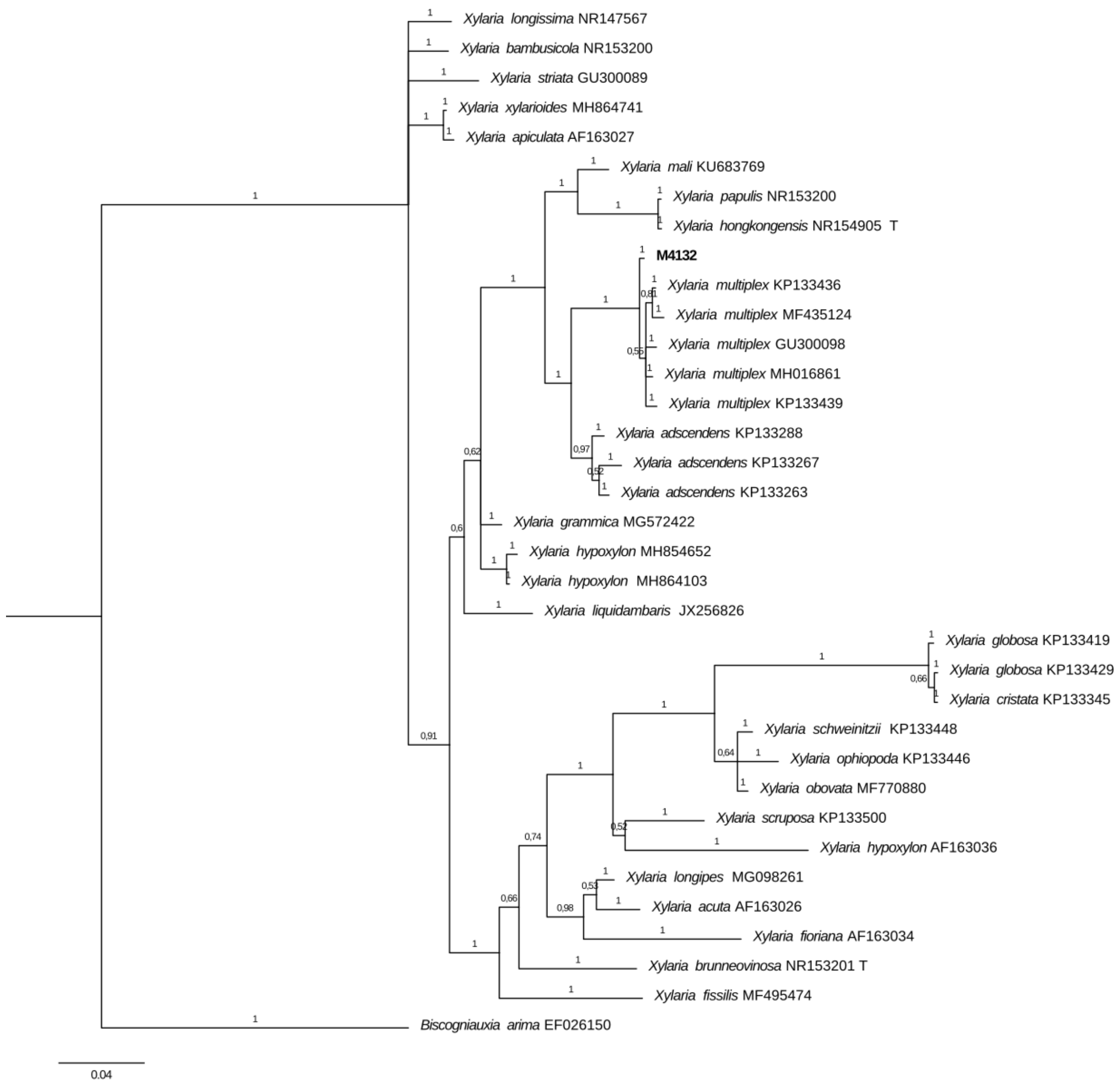

Figure S7. Phylogenetic analysis of *Xylaria* species isolated in this study, with sequences of the closest species deposited in GenBank. The tree was built based on the ITS region sequences using Bayesian Inference.

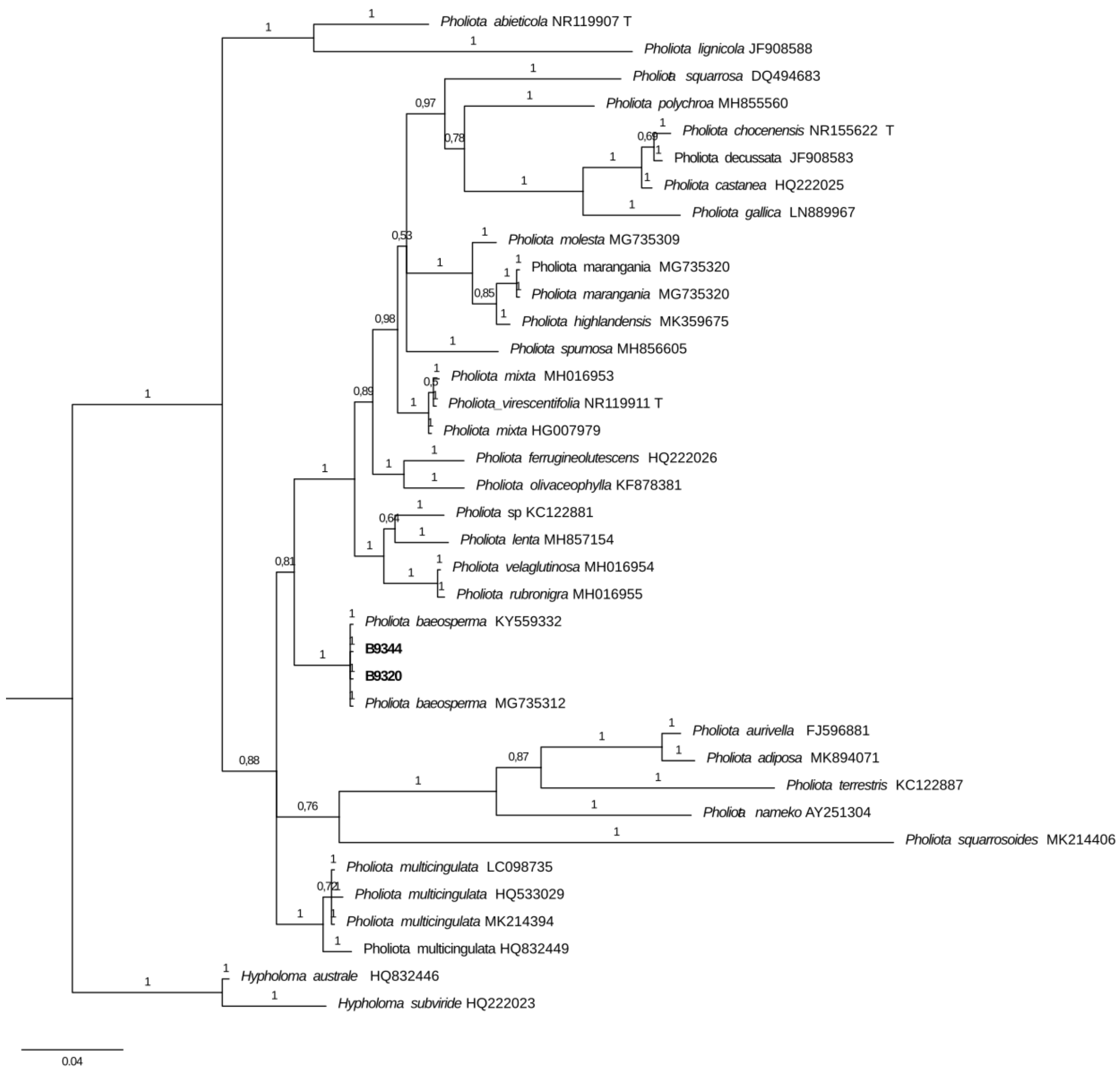

Figure S8. Phylogenetic analysis of *Pholiota* species isolated in this study, with sequences of the closest species deposited in GenBank. The tree was built based on the ITS region sequences using Bayesian Inference.

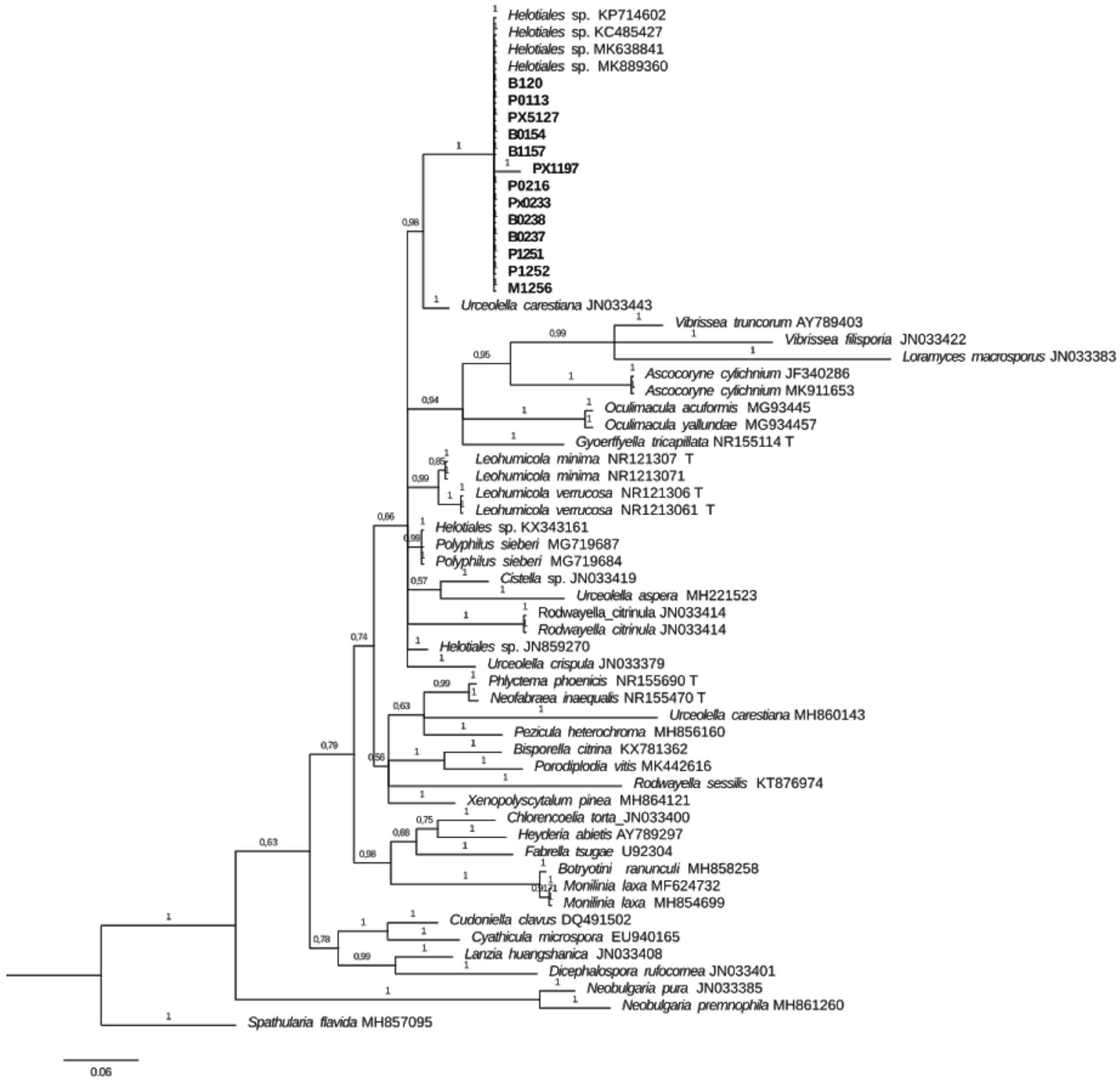

Figure S9. Phylogenetic analysis of *Helotiales* order isolated in this study, with sequences of the closest species deposited in GenBank. The tree was built based on the ITS region sequences using Bayesian Inference

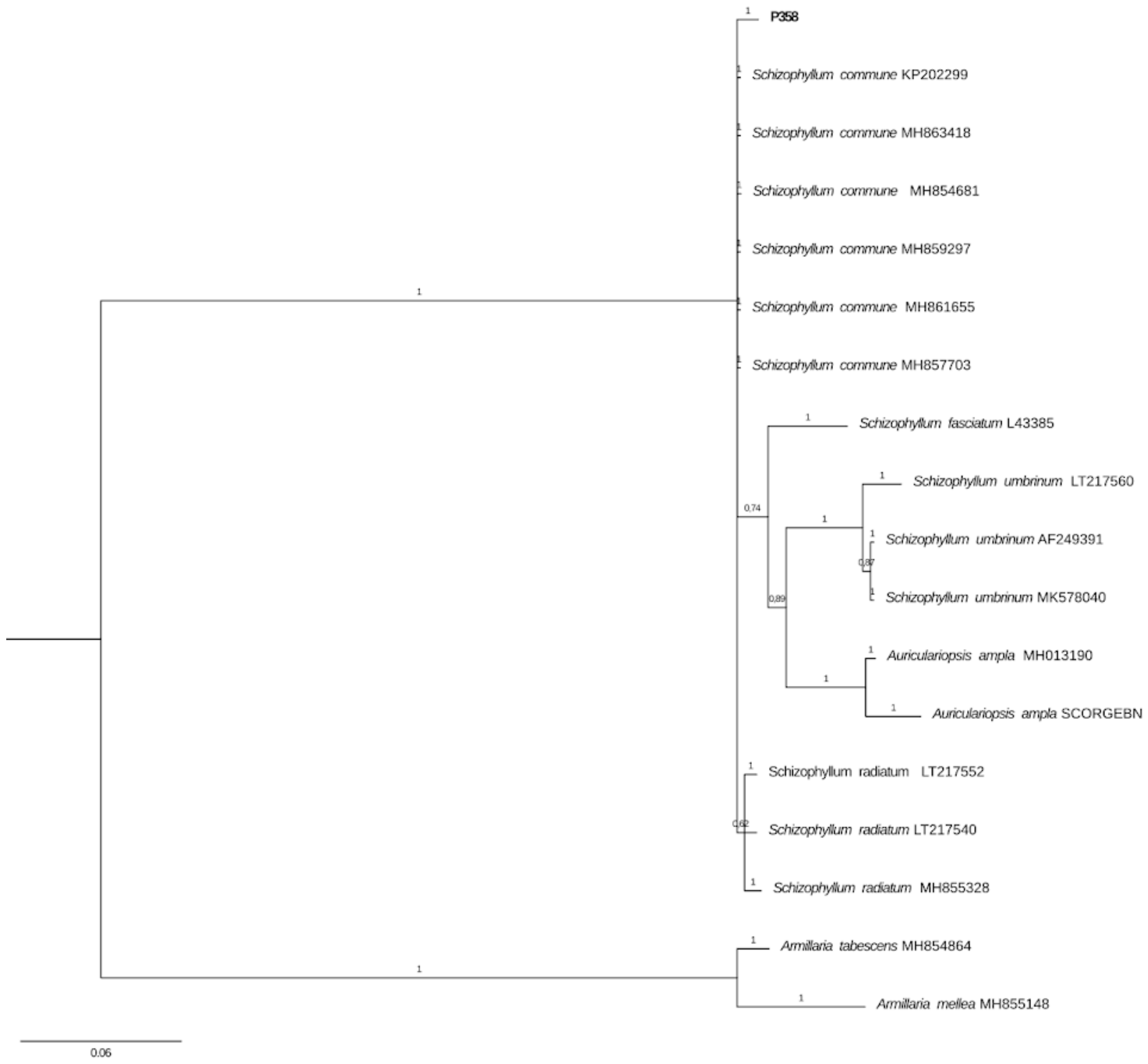

Figure S10. Phylogenetic analysis of *Schizophyllum* species isolated in this study, with sequences of the closest species deposited in GenBank. The tree was built based on the ITS region sequences using Bayesian Inference.

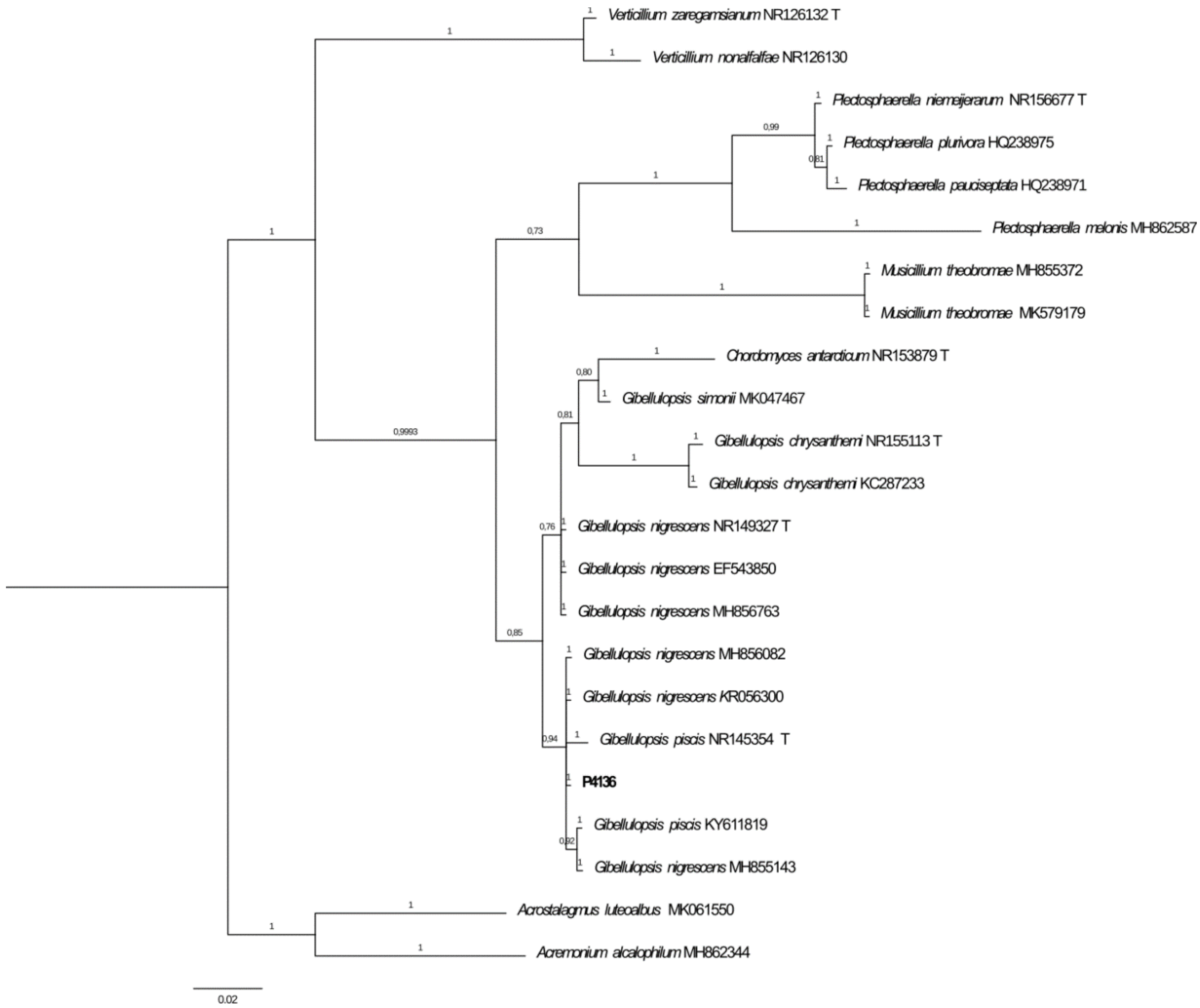

Figure S11. Phylogenetic analysis of *Gibellulopsis* species isolated in this study, with sequences of the closest species deposited in GenBank. The tree was built based on the ITS region sequences using Bayesian Inference.

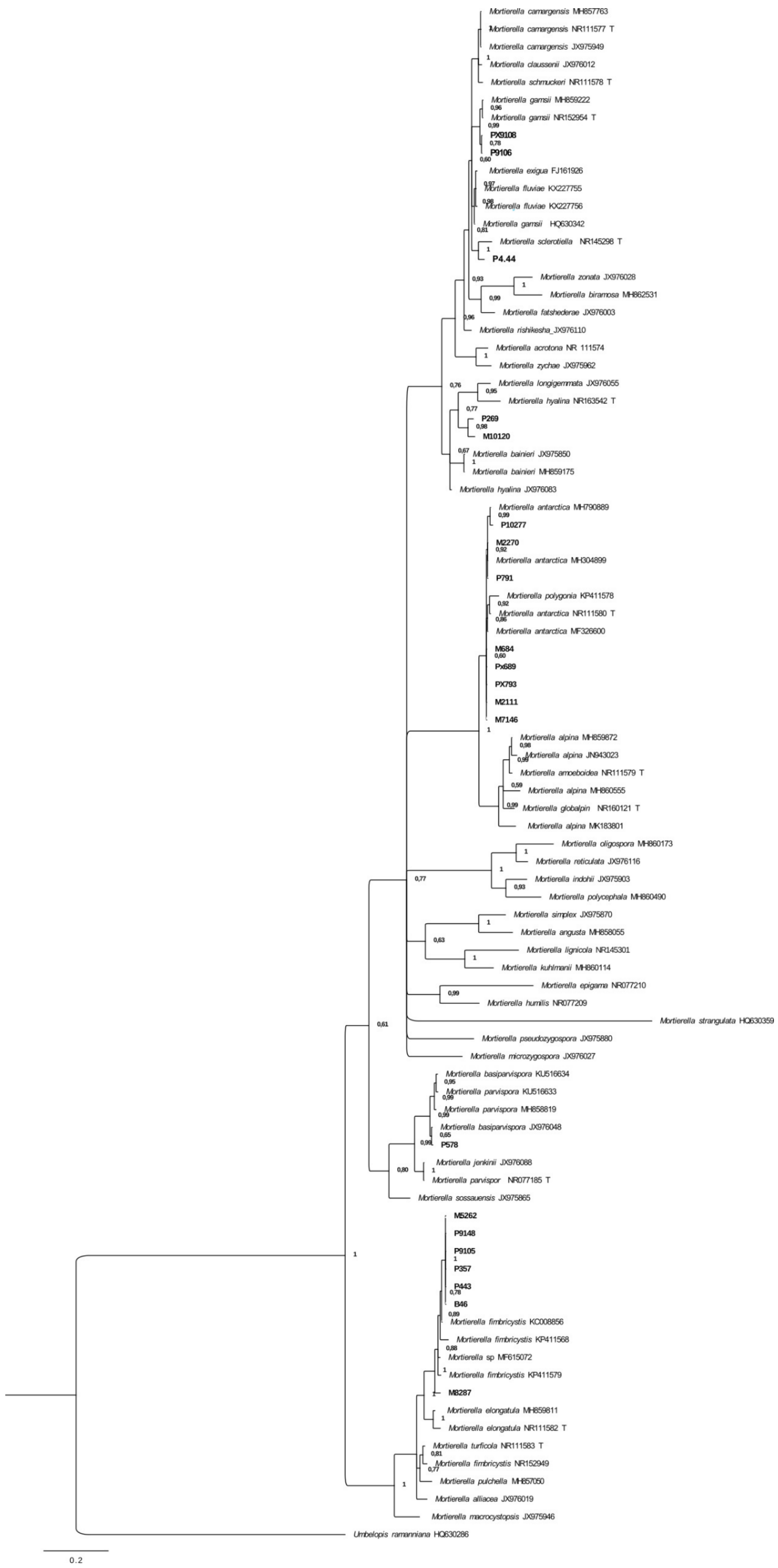

Figure S12. Phylogenetic analysis of *Mortierella* species isolated in this study, with sequences of the closest species deposited in GenBank. The tree was built based on the ITS region sequences using Bayesian Inference.

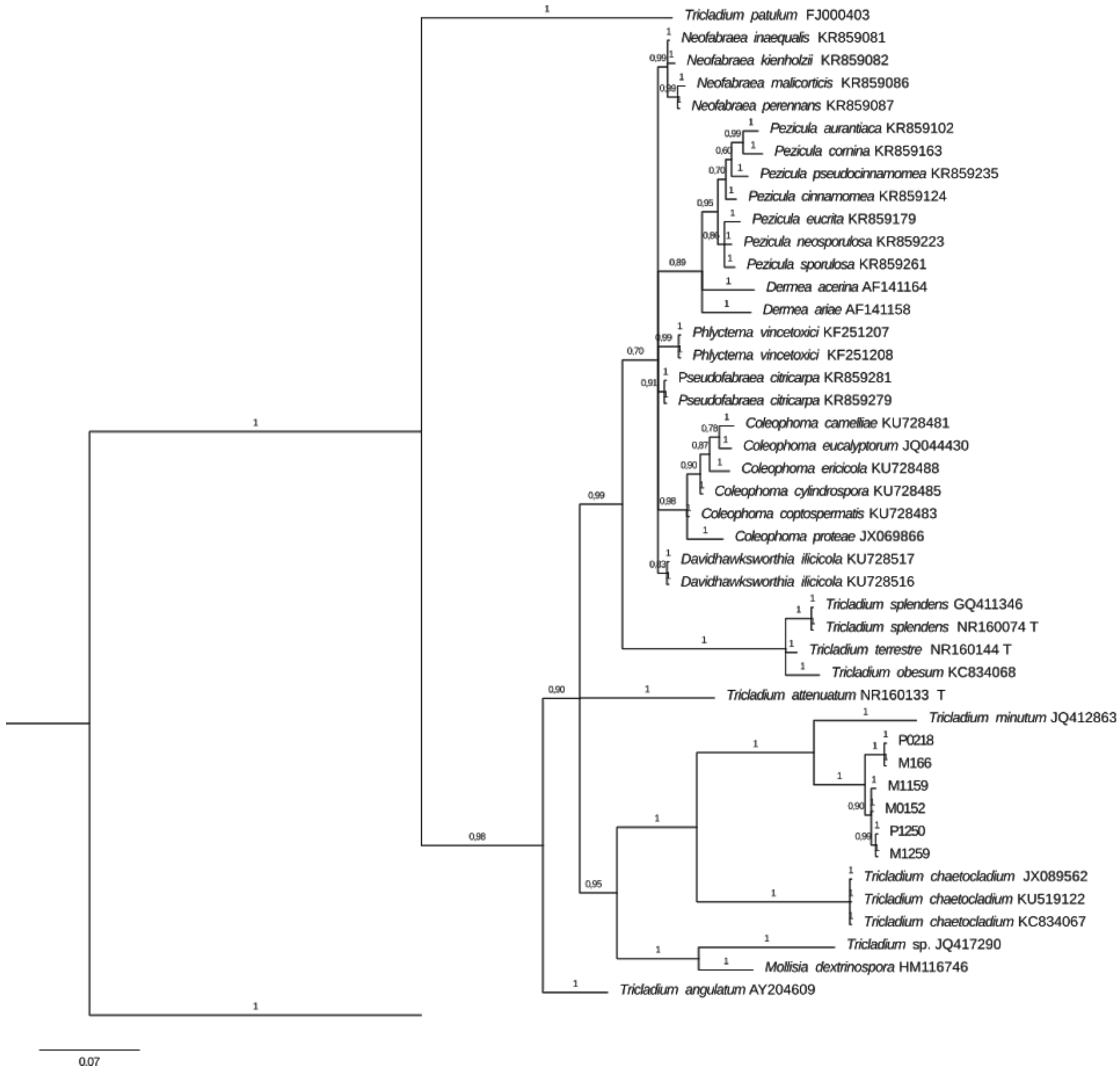

Figure S13. Phylogenetic analysis of *Tricladium* species isolated in this study, with sequences of the closest species deposited in GenBank. The tree was built based on the ITS region sequences using Bayesian Inference.

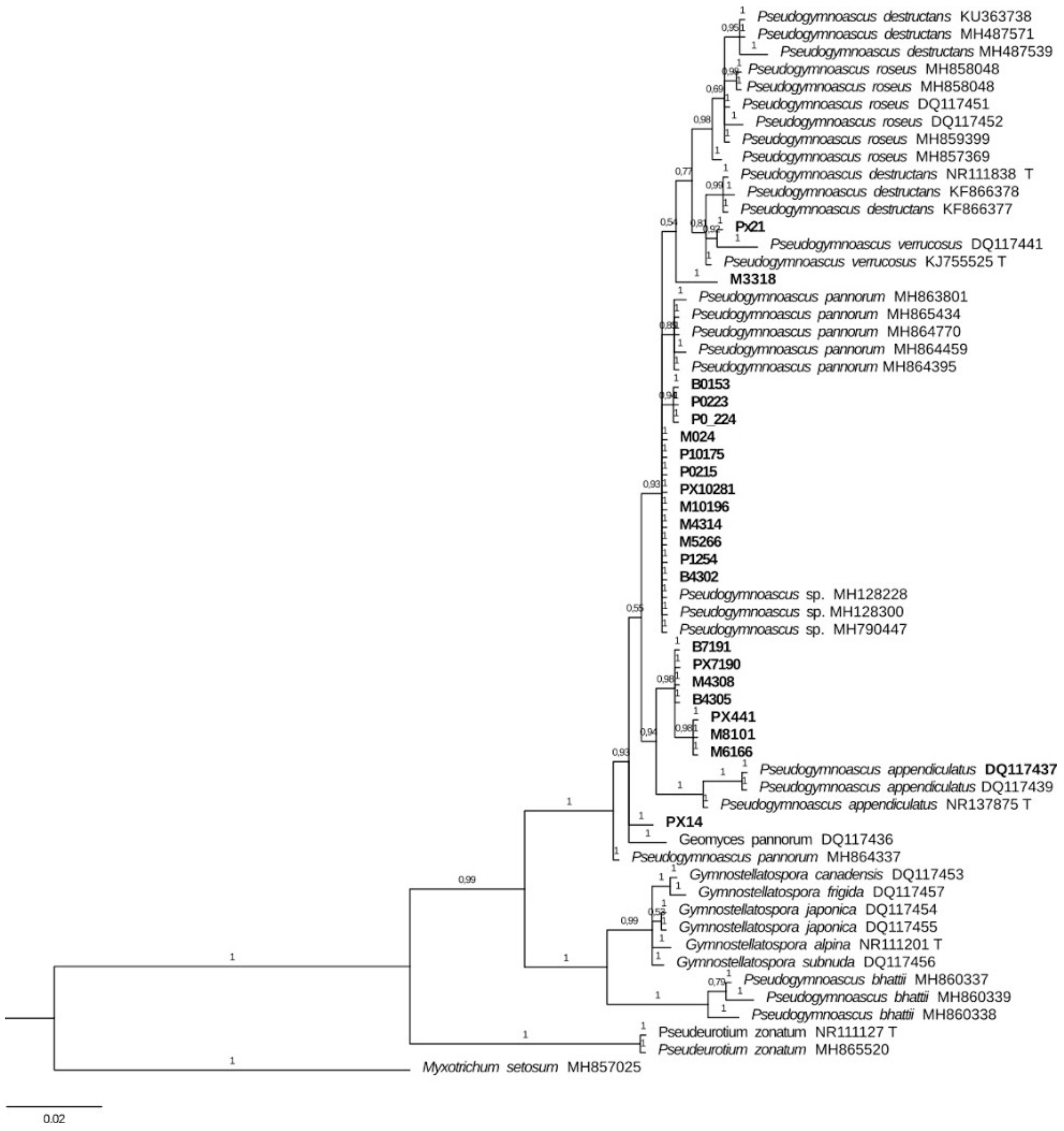

Figure S14. Phylogenetic analysis of *Pseudogymnoascus* species isolated in this study, with sequences of the closest species deposited in GenBank. The tree was built based on the ITS region sequences using Bayesian Inference.

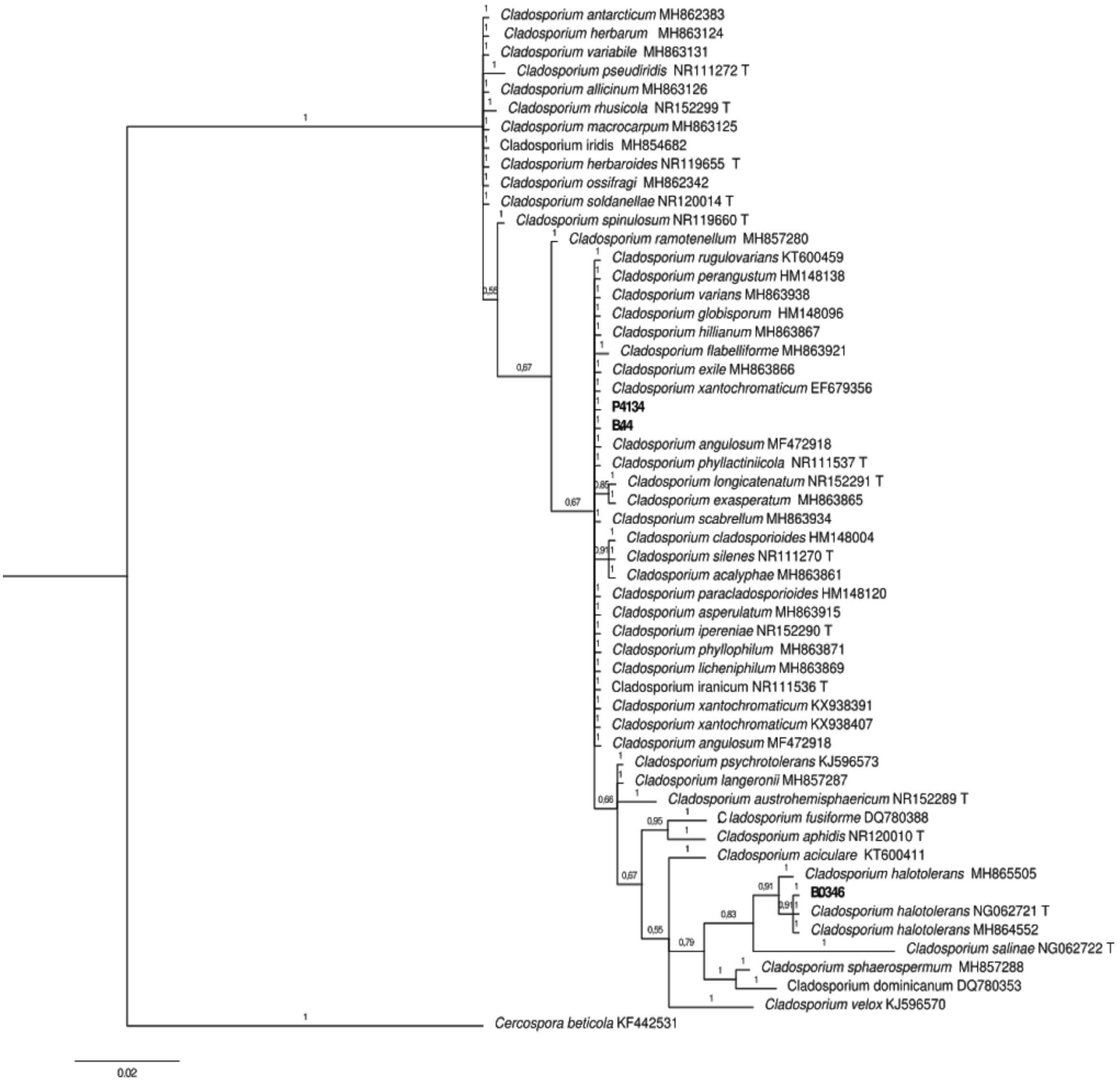

Figure S15. Phylogenetic analysis of *Cladosporium* species isolated in this study, with sequences of the closest species deposited in GenBank. The tree was built based on the ITS region sequences using Bayesian Inference.
